# Identification of New Leaf Rust Resistance Loci in Wheat and Wild Relatives by Array-Based SNP Genotyping and Association Genetics

**DOI:** 10.3389/fpls.2020.583738

**Published:** 2020-11-16

**Authors:** Fizza Fatima, Brent D. McCallum, Curtis J. Pozniak, Colin W. Hiebert, Curt A. McCartney, George Fedak, Frank M. You, Sylvie Cloutier

**Affiliations:** ^1^Ottawa Research and Development Centre, Agriculture and Agri-Food Canada, Ottawa, ON, Canada; ^2^Department of Biology, University of Ottawa, Ottawa, ON, Canada; ^3^Morden Research and Development Centre, Agriculture and Agri-Food Canada, Ottawa, ON, Canada; ^4^Crop Development Centre, University of Saskatchewan, Saskatoon, SK, Canada

**Keywords:** wheat, leaf rust, GWAS, QTN, genotyping array, single nucleotide polymorphism, *Aegilops*, *Triticum*

## Abstract

Leaf rust caused by *Puccinia triticina* is the most widespread rust disease of wheat. As pathogen populations are constantly evolving, identification of novel sources of resistance is necessary to maintain disease resistance and stay ahead of this plant-pathogen evolutionary arms race. The wild genepool of wheat is a rich source of genetic diversity, accounting for 44% of the *Lr* genes identified. Here we performed a genome-wide association study (GWAS) on a diverse germplasm of 385 accessions, including 27 different *Triticum* and *Aegilops* species. Genetic characterization using the wheat 90 K array and subsequent filtering identified a set of 20,501 single nucleotide polymorphic (SNP) markers. Of those, 9,570 were validated using exome capture and mapped onto the Chinese Spring reference sequence v1.0. Phylogenetic analyses illustrated four major clades, clearly separating the wild species from the *T. aestivum* and *T. turgidum* species. GWAS was conducted using eight statistical models for infection types against six leaf rust isolates and leaf rust severity rated in field trials for 3–4 years at 2–3 locations in Canada. Functional annotation of genes containing significant quantitative trait nucleotides (QTNs) identified 96 disease-related loci associated with leaf rust resistance. A total of 21 QTNs were in haplotype blocks or within flanking markers of at least 16 known *Lr* genes. The remaining significant QTNs were considered loci that putatively harbor new *Lr* resistance genes. Isolation of these candidate genes will contribute to the elucidation of their role in leaf rust resistance and promote their usefulness in marker-assisted selection and introgression.

## Introduction

*Triticum aestivum*, commonly known as bread wheat, is an allohexaploid (AABBDD) species, created through the sequential hybridization of three grass species: *T. urartu* (AA), a species (BB) closely related to *Aegilops speltoides* (SS) and *Aegilops tauschii* (DD) (McFadden and Sears, 1946). Genetic diversity bottlenecks such as polyploidization, domestication, and natural and artificial selections have reduced diversity in modern wheat, and consequently increased its vulnerability to diseases, pests and environmental stresses (Tanksley and McCouch, 1997).

Leaf rust caused by *Puccinia triticina* is the most prevalent wheat rust disease, causing tremendous annual yield losses. *Puccinia triticina* attacks the foliage, covering its surface and thus causing loss of photosynthates, dehydration and early defoliation. Genetic resistance combatting yield losses can be categorized into seedling resistance and adult plant resistance (APR). Typically, seedling resistance is controlled by single major effect genes that confer hypersensitive and other responses, causing necrosis and preventing the pathogen from spreading (Dyck and Kerber, 1985). APR occurs at a post-seedling stage and confers either a race-specific or a quantitative race non-specific response (Dyck and Kerber, 1985; Samborski, 1985).

To date, 66 leaf rust resistance (*Lr*) genes have been characterized, six of which, namely *Lr1* (Cloutier et al., 2007), *Lr10* (Feuillet et al., 2003), *Lr21* (Huang et al., 2003), *Lr22a* (Thind et al., 2017), *Lr34* (Krattinger et al., 2009) and *Lr67* (Moore et al., 2015), have been isolated. The majority of the *Lr* genes described to date confer seedling-type resistance. Well-known APR genes include the race-specific *Lr12* (Dyck et al., 1966) and *Lr13* (Dyck et al., 1966) and the race non-specific *Lr34* (Dyck, 1977) and *Lr67* (Hiebert et al., 2010). Of the 66 *Lr* genes designated to date, 37 were identified in *T. aestivum* and *T. turgidum* and 29 originated from progenitors and other wild relative species such as *A. tauschii, A. speltoides, A. neglecta*, and *A. peregrina*, among others (McCallum et al., 2012; USDA, 2017).

The traditional approach for introgressing *Lr* genes into adapted germplasm is by interspecific crossing a donor line to an adapted line followed by backcrossing. Although many *Lr* genes have been described, few are utilized by present-day breeders because they have either been overcome by virulence changes in the pathogen populations, are not in an adapted background or suffer from linkage drag. Modern approaches, such as gene cassettes and genome editing may overcome some of the disadvantages of the crossing method and have been proposed to provide long-lasting broad spectrum resistance (Keller et al., 2016; Arora et al., 2019). However, commercialization of transgenic wheat has not received broad acceptance and introgression via crossing remains commonly used.

Identification of novel sources of resistance in the cultivated and the wild genepools of wheat is expected to contribute to broadening and maintaining the genetic base of leaf rust resistance. Array-based SNP genotyping platforms provide fast and cost-effective access to genetic variation in a diverse germplasm. In wheat, the Illumina’s Infinium iSelect and Affimetrix’s Axiom array technologies enable simultaneous genotyping of 9,000 to 819,571 SNP markers (Cavanagh et al., 2013; Winfield et al., 2016). Genome-wide association studies (GWAS) associate such genotypic data to phenotypic data to identify significant marker-trait associations. To date, numerous quantitative trait loci (QTL) associated with leaf rust resistance in elite cultivars and landraces of bread and durum wheat have been discovered (Aoun et al., 2016; Gao et al., 2016; Riaz et al., 2018). These QTL were identified based on traditional single-locus GWAS models whose inherent limitations, such as failure to capture complex traits controlled by multiple loci, are now widely recognized (Segura et al., 2012). Multi-locus GWAS models overcome these drawbacks by performing a multi-dimensional genome scan, and measuring the effects of multiple SNPs simultaneously to identify small-effect loci for complex traits (Wen et al., 2018). As multi-locus association methodologies are recent, few have been reported in wheat and the potential for multi-locus GWAS covering a diverse range of cultivated wheat and wild relatives remains largely untested.

Here, we performed a GWAS for leaf rust severity and reaction types against six *P. triticina* isolates in a highly diverse germplasm of 385 accessions. The wheat 90 K array was used to capture the genetic variation in cultivated wheats, progenitor species, synthetic hexaploid wheats (SHWs) and wild relatives (Wang et al., 2014). We used one single-locus and seven multi-locus models to identify quantitative trait nucleotides (QTNs) which were mapped on the reference genome, thus validating known loci and identifying new loci to be mined for novel candidate leaf rust resistance genes.

## Materials and Methods

### Plant Materials

A diverse collection of 385 accessions, encompassing 27 different species of cultivated wheats, SHWs, progenitor species and wild relatives were used in this study (Supplementary Table S1). The AB and ABD genomes are represented by 170 accessions representing *T. vavilovii* and several subspecies of *T. turgidum* and *T. aestivum* as well as 65 primary SHWs. The A, B and D genome progenitors (or their closely related species) and the non-domesticated forms of tetraploid wheat comprised 93 accessions of *T. urartu* (A), *T. monococcum* (A^m^), *Ae. tauschii* (D), *Ae. speltoides* (S), as well as *T. turgidum* ssp. *dicoccon* and *dicoccoides* (AB). Another 47 accessions belonged to the following *Aegilops* species: *Ae. bicornis* (S^b^), *Ae. longissima* (S^l^), *Ae. searsii* (S^s^), *Ae. sharonensis* (S^sh^), *Ae. markgrafii* (C), *Ae. comosa* (M), *Ae. umbellulata* (U), *Ae. geniculata* (MU), *Ae. peregrina* (SU), *Ae. triuncialis* (UC/CU), *Ae. columnaris* (UM), *Ae. cylindrica* (DC), *Ae. crassa* (DM/DDM), *Ae. juvenalis* (DMU), *Ae. biuncialis* and *Ae. neglecta* (UM/UMN). The collection also contained six accessions of *T. timopheevii* (A^t^G), five of *T. zhukovskyi* (GAA^m^), and one of *Haynaldia villosa* (V), a related grass species. Overall, the germplasm consisted of 75 diploid, 136 tetraploid, 165 hexaploid and nine accessions that could be either tetraploid or hexaploid. The species names and genome symbols are according to Kimber and Tsunewaki (1988).

### Seed Increase

Seeds were planted and grown under controlled conditions at the Ottawa Research and Development Centre (RDC), Agriculture and Agri-Food Canada (AAFC) (Ottawa, Canada). Depending on their growth habit, the seeds were divided into spring and winter panels. For the spring panel, the growth conditions were 20°C/16 h light, and 16°C/8 h dark. The winter panel was grown under the same conditions for approximately three weeks, i.e., the 4–5 leaf stage, at which time they were transferred to a vernalization cabinet (constant 2°C/12 h photoperiod) for ten weeks to trigger meristem differentiation prior to being returned to the original growing conditions. Seeds harvested from all accessions were used for the greenhouse and field experiments described below.

### Leaf Rust Race-Specific Response

Consecutive inoculations with six *P. triticina* isolates were performed for 360 accessions of the panels (Supplementary Table S2). All tests were performed under controlled greenhouse conditions at the Morden RDC, AAFC (Morden, Canada). Briefly, test lines and the Thatcher and Emerson check lines were sown into fiber trays at a rate of approximately 5 seeds per clump and 3 cm between clumps, which were inoculated with individual *P. triticina* isolates at the two-leaf stage as described by McCallum and Seto-Goh (2006). The isolates tested were 12-3 MBDS, 128-1 MBRJ, 74-2 MGBJ, 11-180-1 TDBG, 06-1-1 TDBG, and 77-2 TJBJ, which represent the prevalent leaf rust race groups across Canada (McCallum et al., 2016). Virulence and avirulence formulas for these isolates are given (Supplementary Table S3). For simplicity, these will be referred to as MBDS, MBRJ, MGBJ, TDBG1, TDBG2, and TJBJ, respectively. Infection type (IT) was rated 12 days post-inoculation using a 0–4 scale (Stakman et al., 1962), where “;” = hypersensitive flecks, “0” = no uredinia or macroscopic sign of infection, “1” = small uredinia with necrosis, “2” = small to medium uredinia with chlorosis, “3” = medium uredinia without chlorosis or necrosis, “4” = large uredinia without chlorosis or necrosis. IT “;” and “0” to “2” were considered resistant, while “3” and “4” were considered susceptible (Long and Kolmer, 1989). The “+” or “−” IT qualifiers indicate larger or smaller than average uredinia, respectively. The “ = ” IT qualifier represents the lower size limit of the uredinia for the IT (Long and Kolmer, 1989). Plants with randomly distributed uredinia of variable sizes, or mesothetic response, were considered resistant and were rated with an “X” IT (Roelfs and Martens, 1988).

For downstream analysis, the IT scores were converted into a 1–9 linear scale, where “0/0;/;” = 1, “;1 = /;1-” = 2, “1-/1/1+” = 3, “;12/1-2-” = 4, “2-/2/2+” = 5, “X” = 6, “3-/3/3+” = 7, “3+4/34” = 8, and “4” = 9 (Supplementary Table S2B). Scores 1–6 were considered resistant and 7–9 susceptible.

### Field Leaf Rust Severity

Phenotyping of rust severity was performed in separate field trials for the spring and winter panels. The spring panel included 213 accessions, of which 20 were SHWs, while the remaining were subspecies of *T. aestivum* and *T. turgidum*. Trials for the spring panel were carried out in Morden, Manitoba, Canada (2016–2019), Ottawa, Ontario, Canada (2017–2019) and Saskatoon, Saskatchewan, Canada (2019). The winter panel comprised 164 diverse *Aegilops* and *Triticum* species, including 115 progenitors and wild relatives, 45 SHWs, and four winter wheat cultivars. Screening for the winter panel was performed in Morden (2017–2019) and in Ottawa (2017 and 2019).

For each panel, year and location, a completely randomized design with two replicates was used, except for the 2016 Morden field trial where a single replicate was used due to the limited seed availability in the first year. For the spring panel, 65 seeds/accession were planted in 1 m-long rows with 20 cm between rows. A mixture of *P. triticina* isolates was inoculated onto spreader rows of susceptible lines Thatcher, Morocco, and Little Club, planted every six test rows in Morden and every ten in Saskatoon. The mixture of isolates comprised more than 50 different virulence phenotypes representing the *P. triticina* population in western Canada identified during the annual virulence survey. In Ottawa, infection relied on natural inoculum but used the same interspersed spreader-row design as Morden with the Morocco spreader. Cultivars Thatcher, Roblin and Eurostar were used as checks and five plots of each were randomly distributed across each replicate.

For the winter panel, ten seeds/accession were planted indoors in early March at both Morden and Ottawa RDCs. At the 3–5 leaf stage, the plants were transferred into vernalization chambers as described above. Approximately ten days after planting the spreader-rows, the vernalized plantlets were transplanted as hills in the field. The cultivar Emerson served as a check. At peak infection and prior to senescence, the flag leaves were rated for leaf rust severity using a modified Cobb’s scale (Peterson et al., 1948).

Leaf rust severity ratings across locations and years were modeled using the R package *Lme4* (Bates et al., 2014) with the following mixed linear model (MLM) equation: *y = lmer(Severity ∼ Location + (1| Genotype) + (1| Year) + (1| Genotype:Location) + (1| Genotype:Year:Location))*. In this model, location was considered a fixed effect, while year, genotype and interaction were considered random. The *lmerTest* package was used to generate an ANOVA-like table for the random effects and calculate *P*-values from the Satterthwaite’s *t*-tests for the fixed effect (Kuznetsova et al., 2017). Best linear unbiased predictors (BLUP) estimates, also known as conditional means, were extracted for the random effects to account for environmental deviations and provide more precise estimates of phenotypic values (Supplementary Table S4) (Mi et al., 2011; Wang et al., 2017).

### Genotyping and SNP Filtering

Young leaf tissue (75–100 mg) from the germplasm grown in growth chambers was sampled at the 4–5 leaf stage. The DNA was extracted using the DNeasy Plant kit (Qiagen, Valencia, CA, United States) and quantified using the Quant-it PicoGreen kit (Thermo Fisher, Waltham, MA, United States). Genotyping was performed using the wheat 90 K array (Illumina, San Diego, CA, United States) on the iScan instrument (Wang et al., 2014).

Genotype calling was performed for the entire collection using the default genotyping module, and separately for the different ploidy levels using the polyploidy module in GenomeStudio software v2.0.4 (Illumina). The tetraploid and hexaploid sets also included the nine accessions of unknown ploidy. The SNP markers with <80% missing data, <5% minor allele frequency (MAF), and >5% heterozygosity were removed. For the polyploid module, markers with >3 clusters were also removed (Hourcade et al., 2019).

### SNP Validation

To validate the filtered SNP dataset, we used an exome dataset obtained from 136 accessions of the panels. First, the position of the SNP markers in the protein coding regions of the Chinese Spring (CS) reference genome v1.0 was obtained by mapping the SNP probe sequences of the wheat Infinium array to “161010_Chinese_Spring_v1.0_gene_sequences_for_exome.fasta” which corresponds to the exome sequence of all the high-confidence annotated genes of the CS reference sequence and their 5 Kb upstream and downstream sequences (Wang et al., 2014; IWGSC, 2018). The SNP probes were aligned to the indexed exome reference sequence using the MEM-BWA algorithm (v0.7.12, http://bio-bwa.sourceforge.net/). As BWA does not accept IUPAC letter codes, two sequences were used for each SNP probe, where one had allele A and the other allele B. Samtools (v1.3, http://samtools.sourceforge.net/) was used to generate and sort the BAM file alignment. The positions of the mapped SNPs were extracted using BBMap (v.38.43 https://sourceforge.net/projects/bbmap/). The mapped SNP probes were filtered using R to remove the misaligned probes, i.e., those for which the A and B allele sequences aligned to different chromosomal positions. The coordinates of the mapped markers were converted to their actual positions on the CS reference genome v1.0 (IWGSC, 2018).

Upon mapping of the SNP markers, the genotyping dataset was re-filtered with the following updated criteria: markers with <80% of χ_i_ missing data, <5% MAF, and >5% heterozygosity were removed, where χ_i_ is the proportion of each sub-genome represented in the germplasm. This less stringent criterion ensures retention of SNPs from underrepresented sub-genomes. The positions of the filtered SNPs from the wheat 90 K array was compared to the variant call results of the exome-sequence data obtained from 136 of the 385 accessions. The exome sequencing data were obtained using the Nimblegen SeqCap EZ wheat exome design (120426_Wheat_WEC_D02, https://sequencing.roche. com/en/products-solutions/by-category/target-enrichment/shar eddesigns.html). Raw reads were mapped to the same exome reference genome using the BWA-Samtools pipeline, and variant calling was performed using Bcftools (v1.3, https:// samtools.github.io/bcftools/bcftools.html). SNPs common between the filtered wheat 90 K array and the exome capture datasets were identified using Bcftools.

### Phylogenetic Relationships, Population Structure and Kinship

To illustrate evolutionary relationships between the species in the collection, the filtered set of SNPs was used to perform a phylogenetic analysis. A maximum likelihood (ML) tree was generated with 1,000 bootstrap iterations using the default parameters of MEGA-CC (Nearest-Neighbor-Interchange heuristic and Tamura-Nei models) (Kumar et al., 2012). The tree was graphically displayed using iTol v3 (Letunic and Bork, 2016). Principal component analyses (PCAs) were performed using the filtered set of SNPs for each ploidy level and the results were displayed using the R package *ggbiplot* (https://github.com/vqv/ggbiplot).

Population structure analyses were carried out using the R packages *LEA* (Frichot and François, 2015) and *PCAdapt* (Luu et al., 2017), as well as the software Admixture v1.3 (Alexander et al., 2009). Both *LEA* and *PCAdapt* estimate structure using PCA-based methods. The proportion of variance explained by each PC was graphically illustrated in the form of scree plots. The “knee” in the scree plot (Cattell’s rule) was used to determine the number of sub-populations. Admixture is an ML-based approach which uses cross-validation to approximate the K number of sub-populations (Alexander and Lange, 2011). Cross-validation errors for K = 2–30 were graphically illustrated using R and the value of K was selected using the rule described above. The approximate number of sub-populations was selected based on the congruity between the plots. The SNMF approach in *LEA* was used to visualize ancestry proportions in the Q matrix through structure plots. The kinship coefficient matrix was generated using Tassel v5.0 (Bradbury et al., 2007).

### Genome-Wide Association Analysis

GWAS was conducted for race-specific response and leaf rust severity rated in the field. For the race-specific response, the converted IT scores for each isolate were considered as individual traits. For leaf rust severity, genotypic and location-specific BLUP estimates were used as phenotypic inputs, where the former summarizes the severity ratings across all locations and years, and the latter represents the severity ratings separately for each location.

GWAS was performed using one single-locus and seven multi-locus models. The MLM in Tassel v5.0 (Bradbury et al., 2007) was used for single-locus association analysis. Here, population structure and kinship were accounted for using Tassel-generated Q matrix for K principle components and the Tassel-generated kinship matrix. The *P*-values were adjusted using the false discovery rate (FDR) (Benjamini and Hochberg, 1995). QTNs with FDR (False discovery rate) -adjusted *P*-values < 0.05 were considered significant.

Of the seven multi-locus models, the six from the R package *mrMLM* (Wen et al., 2018) were mrMLM (Wang et al., 2016), FASTmrMLM (Tamba and Zhang, 2018), FASTmrEMMA (Wen et al., 2018), pLARmEB (Wang et al., 2016), pKWmEB (Ren et al., 2018) and ISIS EM-BLASSO (Tamba et al., 2017). As the *mrMLM* package does not have built-in support for calculating covariates, the Q matrix generated by Admixture and the Tassel-generated kinship matrix were used to account for population structure and kinship, respectively. The seventh multi-locus model, RTM-GWAS, first grouped SNPs into linkage disequilibrium blocks (SNPLDBs) and then utilized a restricted two-stage multi-locus analysis for QTL identification (He et al., 2017). Here, population structure was accounted for by the RTM-generated covariate matrix and kinship and by the Tassel-generated kinship matrix. As with the single-locus MLM, the *P*-values of QTNs from all the multi-locus models used the same FDR-adjusted threshold. Allelic effect of QTNs was determined using the Kruskal–Wallis statistics to test the phenotypic variation of the associated traits between homozygous alleles.

### *In silico* Annotation of Significant Markers

Because only markers that aligned to the exome sequence of the CS reference genome v1.0 were used for the association analyses, all significant QTNs were within or close to high-confidence annotated genes. Transcript IDs of the genes containing the significant QTNs were used to extract the protein products using EnsemblPlants^1^ (Kersey et al., 2016; IWGSC, 2018). For significant SNPLDBs detected by RTM-GWAS that contained multiple SNPs, annotation was carried out for the first and last SNP marker of each SNPLDB.

### Positioning *Lr* Genes and QTNs Onto the Wheat Reference Sequence

Sequences coding for the six previously cloned *Lr* genes were retrieved from GenBank and mapped against the CS reference sequence v1.0 (IWGSC, 2018) using default BLASTn parameters on the GrainGenes website^2^. Through the same exercise, sequences of flanking or co-segregating markers were also mapped onto the reference genome so that a total of 55 of the 66 *Lr* genes were positioned (Supplementary Table S5). A physical map of previously cloned or mapped *Lr* genes was constructed using the R package *KaryoploteR* (Gel and Serra, 2017). Linkage between the QTNs detected and known *Lr* genes, or their markers, was determined using haplotype block analysis. The SNP dataset was split into haplotype blocks using the R package *gpart* (Kim et al., 2019) and pairwise linkage disequilibrium between the SNPs was calculated using Tassel v5.0 (Bradbury et al., 2007). Known *Lr* genes and QTNs within the same haplotype block were considered linked, while the relationship between those in neighboring blocks was determined by comparing *D’* statistics between the blocks.

## Results

### Race-Specific Resistance

IT response against six *P. triticina* isolates (MBDS, MBRJ, MGBJ, TDBG1, TDBG2, and TJBJ) was evaluated in the greenhouse for 360 accessions. Of these, 156, 171, 173, 177, 209 and 206 accessions were resistant (IT rating < 3, linear score < 7) to isolates MBDS, MBRJ, MGBJ, TJBJ, TDBG1 and TDBG2, respectively (Supplementary Table S2 and Supplementary Figure S1). The resistant accessions included 85–131 SHW and cultivated species, 44–56 progenitors and 25–32 wild relatives. Overall, a total of 102 accessions were resistant to all six isolates, and another 153 to at least five isolates.

### Field Resistance

Phenotypic variation across the different environments was modeled. For both spring and winter panels, the year effect explained the smallest proportion of the variance with 2.1% and 0.31% for each panel, respectively, while the largest proportion was accounted for by the genotype effect with 43.0% and 60.2%, respectively (Supplementary Table S6). The *P*-values from Satterthwaite’s *t*-tests were <0.005 for all location effects and the genotype-location interaction explained 16.7% and 21.1% of the variation in the spring and winter panels, respectively. The genotypic and location-specific BLUP estimates were extracted from the models and compared to raw aggregate genotypic and location-specific mean values. A linear relationship was observed between the raw mean values and BLUP estimates (Supplementary Figures S2A,B). However, due to the inherent nature of BLUP estimation to shrink outliers to the mean, the interquartile ranges (Q3-Q1) of location-specific BLUP estimates were smaller than the raw mean values (Supplementary Figures S2C–F).

In the spring panel, 73 accessions were rated resistant (average severity <10%) and 70 were moderately resistant (11–30% average severity) (Supplementary Figure S3A). The majority of the moderately resistant to resistant accessions belonged to the subspecies of *T. turgidum* (Supplementary Table S7A). In the winter panel, respectively, 90 and 38 accessions were rated resistant and moderately resistant; these included 52 progenitors, 36 wild relatives and 12 SHWs (Supplementary Table S7B and Figure S3B). These distributions, however, standardized, were also reflected in the genotypic BLUP estimates calculated for each panel (Supplementary Figures S3C,D).

### SNP Filtering, Mapping and Validation

**FIGURE 1:**
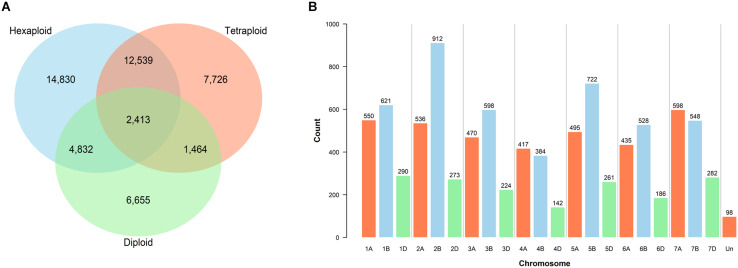
Filtered single nucleotide polymorphism (SNP) markers. **(A)** Shared SNPs between the hexaploid, tetraploid and diploid datasets. **(B)** Distribution of 9,570 filtered and validated SNPs across the chromosomes of the CS reference genome v1.0 (IWGSC, 2018).

A total of 27,418 SNPs from the 385 accessions had a call rate > 80%, of these, 20,501 had a MAF >5% and a maximum heterozygosity <5%. Genotype calling and filtering performed separately for the three ploidy levels yielded 34,614 SNPs in the hexaploid, 24,142 in the tetraploid and 15,364 in the diploid datasets. Shared and private SNPs between the three ploidy levels are illustrated (Figure 1A).

Mapping was performed to locate the position of the 81,587 SNPs of the wheat Infinium assay on the exome sequence of the CS reference genome v1.0 (IWGSC, 2018). A total of 52,550 SNP marker sequences were successfully mapped, of which 43,013 were retained after filtering out the misaligned probes (Supplementary Table S8). Exome-capture sequencing and subsequent variant calling of 136 accessions identified a subset of 27,852 SNPs which belonged to the 43,013 mapped from the array. Re-filtering of the genotyping dataset from the complete germplasm (call rate > 80% of χ_i_, MAF > 5%, and heterozygosity <5%) positioned 12,627 SNPs on the exome reference genome, including 9,570 that were also called using exome capture. Chromosomal assignments of these 9,570 filtered and mapped SNPs illustrate the uneven distribution across genomes (Figure 1B).

### Phylogenetic Relationships and Principal Component Analysis

**FIGURE 2:**
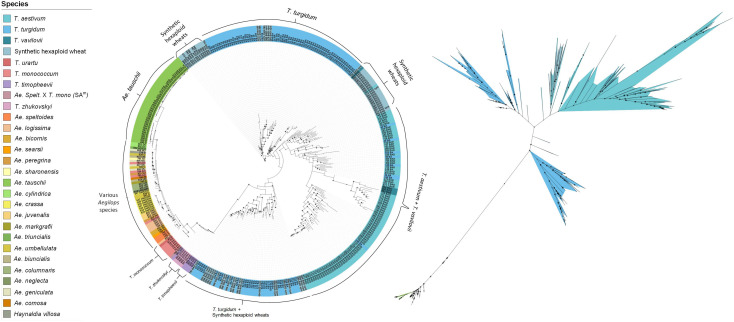
Rooted (left) and unrooted (right) phylogenetic trees illustrating the relationships between species. The tree was generated using the maximum likelihood approach with 1,000 bootstrap iterations. The size of the internal node symbols reflects the bootstrap confidence level and the leaf node labels correspond to the identification number and genome of each accession. The species are color-coded as indicated in the legend.

A ML phylogenetic tree was constructed to illustrate the relationships between the species in the collection. Four main clades were observed (Figure 2). The first consisted of all the *Aegilops* and non-domesticated *Triticum* species, where accessions clustered based on their shared sub-genomes. The second and largest clade comprised accessions with the ABD genome: SHWs, *T. vavilovii* and *T. aestivum* subspecies. The other two clades were primarily a mixture of *T. turgidum* subspecies and SHWs. Ancient tetraploid species *T. turgidum* ssp. *dicoccum* (emmer wheat) and the non-domesticated *T. turgidum* ssp. *dicoccoides* formed one clade, while modern cultivated species, such as *T. turgidum* ssp. *durum* and *T. turgidum* ssp. *carthlicum*, formed the other. SHWs were distributed between these clades based on their tetraploid parent species.

**FIGURE 3:**
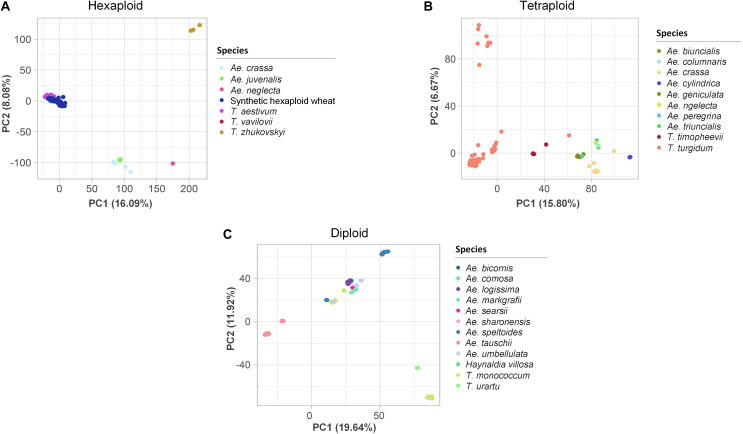
Two-dimensional scatter plots of the first two principal components (PCs) estimated for the **(A)** hexaploid, **(B)** tetraploid, and **(C)** diploid single nucleotide polymorphism datasets. The accessions are colored based on their species. The percentages of the variance explained by each PC are in brackets on the axes.

PCAs were performed to assess the genetic variation at different ploidy levels. In the hexaploid dataset, the first three PCs explained 29.0% of the variation (Figure 3A). Three to four main clusters were observed: the ABD genome species, *T. aestivum* and *T. vavilovii*, formed one cluster, *Ae. crassa* (DM/DDM) and *Ae. juvenalis* (DMU) formed a second closely related cluster, while *Ae. neglecta* (UM/UMN) and *T. zhukovskyi* (GAA^m^) clustered into two distinct groups. Similarly, in the tetraploid dataset, the first three PCs explained 28.3% of the variation (Figure 3B). Here, *T. turgidum* subspecies clustered into three groups, while *T. timopheevii* (A^t^G), *Ae. crassa* and *Ae. cylindrica* (DC) clustered into individual groups. Accessions belonging to species with the U or M sub-genome (*Ae. geniculata*, *Ae. peregrina*, *Ae. triuncialis*, *Ae. biuncialis*, *Ae. columnaris* and *Ae. neglecta*) clustered together. In the diploid dataset, the first three PCs explained 39.9% of the variation. *Ae. tauschii* (D) accessions clustered into two groups, *Ae. speltoides* (S) and *T. monococcum* (A^m^) clustered separately, and nine other species, each represented by few accessions, all clustered as individual groups (Figure 3C). Eight accessions did not cluster with other individuals of their respective species. They were assumed to have been mis-labeled and were removed from the datasets (Supplementary Table S9).

### Genome-Wide Association Analysis

GWAS was performed using IT scores against six *P. triticina* isolates and the leaf rust severity measured in multiple field environments. For each dataset, the population structure was estimated using three tools and the optimal number of sub-populations was selected based on agreement between methods. For IT scores, K = 8 was selected, and for leaf rust severity, K = 8 and K = 6 were selected for the spring and winter panels, respectively (Supplementary Figures S4, S5).

GWAS was conducted using one single-locus and seven multi-locus models, all of which accounted for kinship and population structure. For IT response, the single-locus MLM identified five QTNs for which the proportion of variance explained (*r*^2^) ranged from 6–12% (Supplementary Table S10). Of these, four QTNs were identified for response against the isolate MBDS. The six multi-locus models from *mrMLM* identified a total of 116 unique QTNs across the genome, of which 32 were identified by more than one model and 23 were associated with more than one isolate (Supplementary Table S10). Of note, markers Tdurum_contig18471_456 and IAAV6025 associated with MBDS and Kukri_c12869_154 associated with TDBG1 had *r*^2^ values > 27%, while *r*^2^ values ranged from 1–23% for the remaining QTNs. RTM, the seventh multi-locus model, grouped the SNPs into 7,607 SNPLDBs and identified 15 QTL with *r*^2^ of 4–15%, including eight that had previously been detected by other multi-locus models (Supplementary Table S10). Of the five QTNs identified by single-locus GWAS, four were identified by at least one of the seven multi-locus models.

GWAS for leaf rust severity was conducted separately for the spring and winter panels. MLM identified five significant QTNs (*r^2^* = 18–24%), all of which were associated with leaf rust severity in Morden and located in the D sub-genome (Supplementary Table S11). In the spring panel, *mrMLM* identified 85 unique QTNs (*r^2^* = 1–22%) associated with leaf rust severity, of which 30 were identified by more than one model and 57 were location-specific (Supplementary Table S11). In the winter panel, 38 QTNs were identified including 10 by more than one model and one at both Morden and Ottawa locations (Supplementary Table S11). Marker wsnp_Ex_c6548_11355524 on 5B explained the highest proportion of the variance (40%), while *r*^2^ of the remaining QTNs ranged from 2–24%. RTM identified 37 QTL in the two panels, including seven that were also identified by other multi-locus models (Supplementary Table S11). Overall, five QTNs associated with leaf rust severity were also associated with race-specific IT response against at least one isolate. The number of QTNs identified by each model, for both, IT response and leaf rust severity, are shown in Table 1. For each phenotypic dataset, the multi-locus model mrMLM identified the highest number of QTNs, while the single-locus model MLM identified the fewest. The eight GWAS models were further compared based on *r*^2^, FDR-adjusted *P*-values and the number of common QTNs (Supplementary Figures S6, S7).

**TABLE 1:** Number of QTNs or SNP linkage disequilibrium blocks identified by each statistical model for infection type (IT) and leaf rust (LR) severity.

**Model**	**IT**	**LR severity spring panel**	**LR severity winter panel**	**Total**
**Single-locus**				
MLM	5	1	4	10
**Multi-locus**				
FASTmrEMMA	14	14	1	29
FASTmrMLM	33	20	8	61
ISIS EM-BLASSO	30	22	14	66
mrMLM	54	36	10	100
pKWmEB	38	30	10	78
pLARmEB	41	25	13	79
RTM	15	21	16	52
**Total**	230 (124)	169 (104)	76 (53)	

*Values in parentheses indicate the number of non-redundant loci, i.e., loci identified by at least one statistical model.*

### Functional Annotation

**FIGURE 4:**
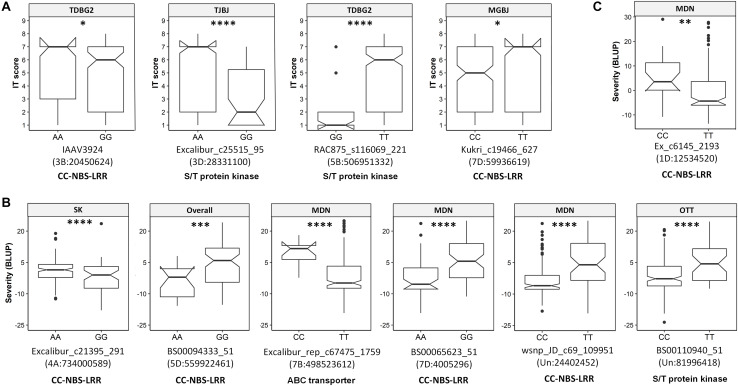
Boxplots showing significant allelic effects for quantitative trait nucleotides (QTNs) present within genes encoding known disease resistance protein types. **(A)** QTNs associated with race-specific IT response. **(B)** QTNs associated with leaf rust severity in the spring panel. **(C)** QTNs associated with leaf rust severity in the winter panel. Labels at the top of each boxplot show the associated trait, i.e., the isolate for **(A)** or the overall mean or location-specific leaf rust severity **(B, C)**. QTNs, chromosomal locations and annotation are indicated below each plot. Locations are Morden (MDN), Ottawa (OTT) and Saskatoon (SK). Kruskal–Wallis significance levels “^∗^,” “^∗∗^,” “^∗∗∗^,” and “^****^” correspond to *P*-value ≤ 0.05, 0.01, 0.001, and 0.0001, respectively.

The transcript IDs of the genes within 5 Kb of one or more QTN were extracted along with their functional annotation. Between 79–85% of the QTNs associated with race-specific IT response and leaf rust severity were successfully annotated for gene function (Supplementary Table S12). A total of 46 loci associated with race-specific response and 50 associated with LR severity (37 in the spring panel and 13 in the winter panel) were within 5 Kb of genes coding for known plant disease resistance proteins such as CC-NBS-LRR, F-box-like domain-containing proteins, proteins with kinase domains, zinc finger-types and ABC transporter proteins, among others (Supplementary Table S12). A combined total of 53 QTNs with *r^2^* > 5% were located within plant disease resistance genes (Table 2). For each of these, Kruskal–Wallis tests were performed to test the statistical significance in phenotypic values of the alternate alleles. Significant allele-phenotype differences (*P*-value < 0.05) were obtained for 35 of the 53 QTNs or SNPLDBs (Table 2), where the favorable alleles came from the domesticated *T. aestivum* and *T. turgidum* species as well as the wild relative species (Supplementary Figure S8). Phenotypic variation for 11 of these significant QTNs present within CC-NBS-LRR, ABC-transporter and protein kinase domains are illustrated (Figure 4). Such QTNs were considered strong candidate genes as their function and allelic variation are congruent.

**TABLE 2:** Chromosomal location and functional annotation of quantitative trait nucleotides (QTNs) or linkage disequilibrium blocks (LDBs) associated with race-specific infection type and leaf rust severity.

**QTN/LDB**	**Chr**	**Position**	**Trait**	**Model**	**R^2^**	**KW**	**R allele**	**Co-located *Lr* gene^†^**	**Gene annotation**
**Infection type**									
IAAV6025	1B	211312483	MBDS	FASTmrEMMA	28.0	****	G		PC-Esterase, PMR5 N-terminal domain
				MLM	5.9				
Kukri_rep_c115699_270	2D	39829875	TDBG2	FASTmrEMMA	5.8	**	G	*Lr15*	Serpin superfamily
tplb0052b23_2493	2D	621964724	TDBG1	FASTmrEMMA	6.2	Ns	−		LRR domain, NBS, CC domain
				pKWmEB	5.3				
RFL_Contig3121_1979	2D	648470008	MBDS	pKWmEB	5.8	Ns	−	*Lr54*	LRR domain, S/T-protein kinase
Tdurum_contig18471_456	3A	75030422	MBDS	mrMLM	34.6	****	G		F-box-like domain
D_GCE8AKX02HMJXL_374	3B	15138756	MBDS	MLM	12.6	****	A	*Lr27*	Jacalin-like lectin domain
				pKWmEB	5.4				
IAAV3924	3B	20450624	TDBG2	pKWmEB	6.8	*	G		LRR domain, NBS, CC domain
Kukri_c12869_154	3B	130647769	TDBG1	mrMLM	27.9	***	C		F-box-like domain
Excalibur_c25515_95	3D	28331100	TJBJ	ISIS EM-BLASSO	7.7	****	G	*Lr32*	S/T-protein kinase
				pKWmEB	6.6				
				pLARmEB	5.2				
				FASTmrMLM	5.2				
D_contig10567_587	3D	141408492	MBDS	MLM	6.2	****	C		Glycoside hydrolase
D_contig29825_215	4D	82020798	TDBG1	FASTmrMLM	11.2	ns	−		LRR domain, NBS, CC domain
				pLARmEB	8.2				
			TDBG2	pKWmEB	11.0				
				pLARmEB	5.1				
RAC875_c9984_1003	5A	585458451	TDBG1	TDBG1	8.7	****	A		P-loop NTPase, Kinesin motor domain
wsnp_BJ224975A_Ta_2_2	5A	588737306	TDBG2	RTM	5.4	Ns	−		Protein kinase, ATP binding site
Kukri_c17055_189	5A	588742167	TDBG2	RTM	5.4	****	T		P-loop NTPase, ABC transporter
RAC875_s116069_221	5B	506951332	TDBG2	pKWmEB	7.2	****	G		Serine/threonine-protein kinase
D_contig18780_204	5D	486259068	TDBG2	FASTmrEMMA	9.3	*	A		LRR domain, NBS, CC domain
wsnp_Ra_c3766_6947263	6B	151130562	MBDS	RTM	6.6	****	A		ZTL, PAS domain, beta-propeller, F-box-like
Kukri_c39321_112	6B	151131531	TDBG2	pKWmEB	11.3	****	C		ZTL, PAS domain, beta-propeller, F-box-like
			TJBJ	pKWmEB	6.7				
			MBDS	RTM	6.6				
Kukri_c3664_1071	6D	10910854	MGBJ	mrMLM	6.8	*	G		P-loop NTPase, AAA+ ATPase domain
Ex_c54863_29	7B	561748617	TDBG2	RTM	14.7	****	C		Zinc finger, TAZ/FYVE/PHD-type,
BS00063208_51	7B	637618402	MGBJ	mrMLM	5.4	**	T		LRR domain
Kukri_c19466_627	7D	59936619	MGBJ	pKWmEB	18.8	*	C		LRR domain, NBS, CC domain
**Leaf rust severity in the spring panel**									
wsnp_Ex_c3372_6195001	1A	257573729	SK	ISIS EM-BLASSO	6.4	***	T		LRR domain
				RTM	5.8				
Excalibur_c33567_363	1A	427819541	SK	mrMLM	17.6	ns	−		Zinc finger, FYVE/PHD-type
wsnp_Ex_rep_c67474_66076379	1A	591093391	OTT	pLARmEB	9.2	ns	−		P-loop NTPase, Armadillo-type fold
				FASTmrMLM	8.8				
Kukri_c55909_1109	2B	770681399	OTT	FASTmrMLM	7.9	****	C		WD40-repeat-containing domain
CAP8_c3568_256	3A	724200935	SK	mrMLM	10.0	ns	−		Alpha/Beta hydrolase fold
				pLARmEB	6.8				
Excalibur_c21395_291	4A	734000589	SK	FASTmrMLM	5.0	*	G	*Lr28*	LRR domain, NBS, CC domain
RAC875_c2099_2066	4B	670439103	Overall	pKWmEB	5.1	****	T		Zinc finger, FYVE/PHD-type
Tdurum_contig10128_593	5A	48464957	OTT	RTM	9.7	*	C		Papain-like cysteine peptidase
Excalibur_rep_c67473_320	5B	506789600	MDN	pKWmEB	5.1	ns	−		P-loop NTPase, AAA+ ATPase domain
BS00094333_51	5D	559922461	Overall	FASTmrMLM	9.6	***	A	*Lr1*	LRR domain, NBS, CC domain
				FASTmrEMMA	8.4				
				ISIS EM-BLASSO	8.4				
				pKWmEB	7.6				
				pLARmEB	6.5				
BS00037002_51	6A	2972683	MDN	FASTmrEMMA	5.8	Ns	−		F-box-like domain
RAC875_c68525_284	6B	657946526	SK	mrMLM	13.3	*	G		Zinc finger, FYVE/PHD-type
BobWhite_rep_c66074_232	6B	706118869	Overall	FASTmrEMMA	5.1	Ns	−		LRR domain
Kukri_c58096_480	6B	712390096	MDN	RTM	14.7	***	T		Papain-like cysteine peptidase
CAP7_c2923_366	6D	129784133	MDN	ISIS EM-BLASSO	11.8	***	T		Plant lipoxygenase, PLAT/LH2 domain
				FASTmrEMMA	11.6				
CAP11_c5372_271	6D	465742757	MDN	RTM	10.4	Ns	−		LRR domain, F-box-like domain
Excalibur_rep_c67475_1759	7B	498523612	MDN	mrMLM	5.9	****	T		P-loop NTPase, PDR ABC transporter
				ISIS EM-BLASSO	5.7				
tplb0021f14_1700	7B	653898244	Overall	mrMLM	9.5	Ns	−		Serine/threonine-protein kinase
Excalibur_rep_c74234_183	7B	655037014	Overall	ISIS EM-BLASSO	5.1	ns	−		Serine/threonine-protein kinase
BS00065623_51	7D	4005296	MDN	pKWmEB	18.4	****	A		LRR domain, NBS, CC domain
				FASTmrEMMA	11.2				
D_GDS7LZN02FSYZC_227	7D	58491641	OTT	pLARmEB	5.1	ns	−		LRR domain, NBS, CC domain
wsnp_JD_c69_109951	Un	24402452	MDN	FASTmrMLM	13.5	****	C		LRR domain, NBS, CC domain
				pLARmEB	11.0				
				ISIS EM-BLASSO	6.4				
BS00110940_51	Un	81996418	OTT	pKWmEB	7.9	****	C		Serine/threonine-protein kinase
**Leaf rust severity in the winter panel**									
BS00067436_51	1A	578204373	MDN	FASTmrMLM	12.7	**	G		Glycoside hydrolase
Ex_c6145_2193	1D	12534520	MDN	RTM	6.5	**	T		LRR domain, NBS, CC domain
				mrMLM	7.8				
Kukri_c59403_339	2D	75001895	MDN	MLM	19.7	Ns	−		WD40-repeat-containing domain
Excalibur_c3862_837	3B	245969143	MDN	FASTmrMLM	8.5	****	A		Peroxidase
BS00022555_51	5B	435769407	OTT	pKWmEB	14.0	ns	−		F-box-like domain, beta-propeller
wsnp_Ex_c6548_11355524	5B	439725143	MDN	ISIS EM-BLASSO	21.7	ns	−		WRKY domain
				pKWmEB	22.8				
				mrMLM	40.1				
Kukri_c855_2107	7A	708138675	MDN	ISIS EM-BLASSO	7.3	ns	−		Zinc finger, CCCH-type
D_contig28902_391	7D	456495802	Overall	ISIS EM-BLASSO	5.0	*	A		F-box-like domain

Only QTNs/LDBs located within disease resistance-related proteins explaining greater than 5% of the phenotypic variation are shown. **^†^***Lr* genes present within the same or neighboring haplotype block, or *Lr* genes mapped using flanking markers. Chr, Chromosome; MDN, Morden; OTT, Ottawa; SK, Saskatoon; R allele, allele for resistance; KW, Kruskal–Wallis test significance level where “ns,” “*,” “**,” “***,” and “****” correspond to not-significant and *P*-value ≤ 0.05, 0.01, 0.001, and 0.0001, respectively.

### Comparing Associated Loci With Previously Reported *Lr* Genes

**FIGURE 5:**
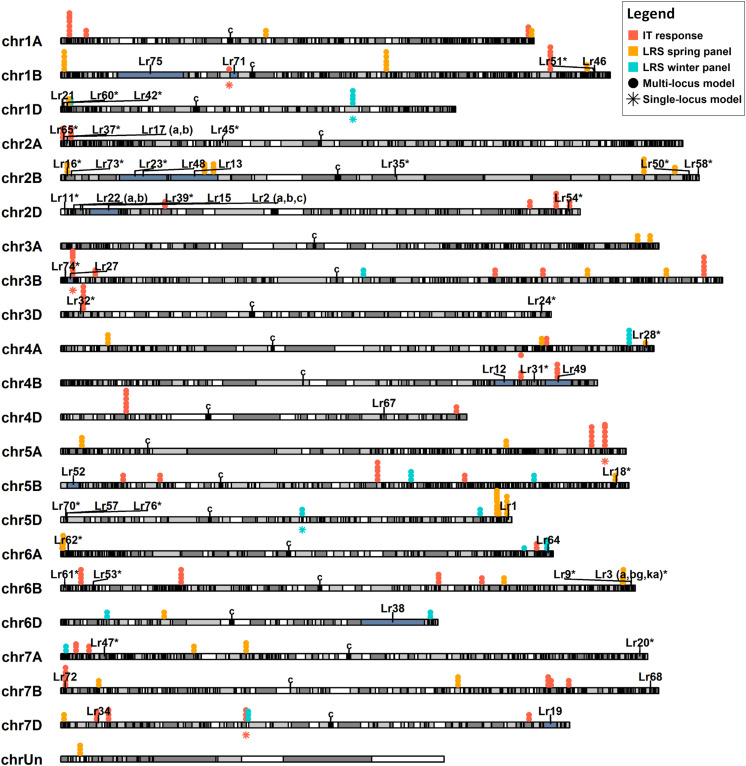
Physical map illustrating the position of 55 known leaf rust resistance genes (*Lr*) and quantitative trait nucleotides (QTNs) associated with leaf rust severity (LRS) and infection type (IT) against six leaf rust races. The positions of the previously cloned *Lr* genes are indicated by a single vertical line on the chromosome. Regions shaded in gray indicate linkage disequilibrium blocks, while those shaded in blue indicate the location of the proximal and distal markers of mapped *Lr* genes. *Lr* genes previously mapped with a single marker are indicated with an asterisk (*). For simplicity, QTNs associated with race-specific IT are shown in orange and those associated with LRS in the spring and winter panels are color-coded in yellow and blue, respectively. Solid dot (•) QTNs indicate association through a multi-locus model while star (⋆) QTNs were identified with the single-locus model MLM. Centromeres are denoted with a “c” symbol. Only QTNs identified by more than one model are shown.

To identify novel putative disease resistance loci, the physical positions of the QTNs identified were compared to the positions of the 66 previously reported *Lr* genes (Supplementary Table S5). All QTNs and *Lr* genes, except for *Lr10, Lr14 (a,b), Lr25, Lr26, Lr29, Lr30, Lr36, Lr44, Lr56*, *Lr59*, and *Lr66*, were physically mapped on the CS reference genome v1.0 (IWGSC, 2018). The positions of these mapped *Lr* genes and the IT and leaf rust severity QTNs identified herein by at least two models are illustrated (Figure 5).

Of the *Lr* genes mapped using both proximal and distal flanking markers, markers for *Lr12*, *Lr13*, *Lr15*, *Lr19*, *Lr27*, *Lr28*, *Lr49*, *Lr64*, and *Lr75* co-located with 13 of the QTNs identified (Supplementary Table S13). These include seven QTNs associated with leaf rust severity and six with IT response. Haplotype black analysis was used to evaluate the relationships between the QTNs detected and the *Lr* genes mapped using gene sequences or single genetic markers. A total of 2113 haplotype blocks (*D’* ≥ 0.5) were obtained, with an average block size of 4.9 MB. Two QTNs, BS00094333_51, associated with leaf rust severity, and D_GDS7LZN02F1Q5F_180, with IT caused by isolates TDBG1, MGBJ and TJBJ, were in the same haplotype blocks as the cloned genes *Lr1* and *Lr34*, respectively, while three co-located in the same blocks as genetic markers of *Lr16*, *Lr32* and *Lr73* (Supplementary Table S13). Apart from this, another three QTNs were in neighboring blocks of the markers linked to *Lr18* and *Lr54.* Pairwise linkage analysis between these blocks resulted in mean *D’* statistics ranging from 0.44 to 0.66. Overall, Kruskal–Wallis tests identified significant allele-phenotype differences (*P*-value < 0.05) for 14 of the 21 QTNs mapping near positions of known *Lr* genes (Table 2, Supplementary Table S13).

## Discussion

*Puccinia triticina* populations are constantly evolving, as exemplified by the presence of more than 70 races detected in North America each year (Ellis et al., 2014). This can quickly render the deployed *Lr* genes ineffective. Identification of novel sources of disease resistance is necessary to stay ahead in this plant-pathogen evolutionary arms race and to maintain disease resistance in crops. The ability to detect novel *Lr* genes through marker-based association studies depends greatly on the phenotypic and genetic variation present in the germplasm. The majority of the GWAS in wheat are based on elite cultivars, breeding lines or landraces sourced from breeding programs, genebanks or private seed collections, mainly because introgression into adapted germplasm is easier and faster from the primary genepool as compared to more distant germplasm (Gao et al., 2016; Riaz et al., 2018). These collections, although geographically adapted, often provide limited genetic diversity due to the domestication and selective breeding bottlenecks. Conversely, ancestors and wild relatives of wheat lack adaptation traits for agriculture, but are a rich source of genetic variation, accounting for 44% of the *Lr* genes identified to date (McCallum et al., 2012; USDA, 2017). In the past century, research to identify and transfer resistance genes from wild relatives was laborious, lengthy and focused on one gene at the time. Recent developments in genotyping technologies and the release of the wheat reference genome are enabling high throughput identification of new resistance genes regardless of the genepool, and thus accelerating their gene cloning (IWGSC, 2018; Arora et al., 2019). Here, we described an efficient method to identify new *Lr* gene loci and candidate genes from many *Triticum* and *Aegilops* species using an array-based SNP genotyping platform and eight GWAS models. Through this approach, we identified a total of 50 and 46 disease-related QTNs associated with field leaf rust severity and IT response against six *P. triticina* isolates, respectively, several of which were located near known *Lr* genes and others were linked to putative new ones. The QTNs identified in this study provide the framework for investigating novel and effective *Lr* genes from this diverse germplasm and for cloning known *Lr* genes.

### Genetic Diversity

Bread wheat, an allohexaploid species, comprises an estimated 17 billion nucleotides, more than 85% of which is repetitive DNA (IWGSC, 2018). Array-based SNP genotyping platforms provide a quick and cost-effective opportunity to survey whole genomes of a large number of samples. We used the wheat 90 K array to genotype a diverse collection of 385 accessions. A total of 34.1% of the SNPs were shared between the hexaploid and tetraploid datasets, similar to a previous report of 33.9% (Wang et al., 2014). The high percentage of shared SNPs is indicative of the extensive gene flow from the tetraploid ancestors to hexaploid wheat (Dvorak et al., 2006). Because nearly half of the diploid accessions were *Ae. tauschii*, the D genome donor of hexaploid wheat, the total of 7,243 (47.2%) of shared SNPs between the diploid and hexaploid datasets also agrees with the gene flow between these species. Mapping against the CS exome sequence and subsequent comparison with exome capture data identified 9,570 SNPs, from which the B (45.1%), A (36.6%) and D (17.3%) sub-genome distribution compared to several previous reports (Wang et al., 2014; Daba et al., 2018; Pont et al., 2019).

### Structure Analysis

Relationships between the 27 species in the collection were explored using phylogenetic tree analysis. Four major clades were observed, clearly separating the wild species from the *T. aestivum* and *T. turgidum* subspecies. *T. aestivum* ssp. *spelta*, hypothesized to have emerged from hybridization between *T. aestivum* and *T. turgidum* ssp. *dicoccum* (Blatter et al., 2004; Pont et al., 2019), was observed to cluster among the *T. aestivum* subspecies, separately from all *T. turgidum* ssp. *dicoccum* and *dicoccoides.* SHWs created by crossing tetraploid *T. turgidum* with diploid *Ae. tauschii*, were distributed between the two tetraploid clades based on the genetic characterization of their tetraploid parent. For example, 12 SHW accessions created by crossing the durum wheat cultivar ‘Langdon’ with different *Ae. tauschii* accessions, clustered with ‘Langdon’ in the modern tetraploids clade. Similarly, multiple SHWs created by crossing wild emmer wheat accessions PI113961 and PI355465 with *Ae. tauschii*, clustered with their tetraploid parents in the ancient tetraploid clade. Similar studies using SNP, SSR and AFLP markers have reported the genetic diversity of SHWs to clearly reflect the sub-species, geographical origin and morphological traits of their tetraploid parent, possibly due to the fact that it contributed two-third of their genome (Lage et al., 2003; Dreisigacker et al., 2008; Bhatta et al., 2018).

The major clade of wild relatives was separately analyzed to highlight the relationships between the species (Supplementary Figure S9). With the exception of *Ae. sharonensis*, clustering of the *Aegilops* species of the *Sitopsis* section was consistent with previous studies, where *Ae. speltoides* ssp. *speltoides* and *Ae. speltoides* ssp. *ligustica*, formed one clade and *Ae. longissima*, *Ae. bicornis* and *Ae. searsii* formed the other (Bahrman et al., 1988; Sasanuma et al., 1996; Miki et al., 2019). The majority of the *Triticum* species with an A genome also grouped together, where accessions of *T. zhukovskyi* were clustered closer to their tetraploid ancestor *T. timopheevii* (Dvorak et al., 1993). The unique amphiploid EKC22_RL5347 resulting from a cross between *Ae. speltoides* (S) and *T. monococcum* (A^m^) also clustered with the A-genome species. The close relationship between *Ae. crassa* (DM or DDM) and *Ae. juvenalis* (DMU) species was also expected because they both share a D and an M genomes (Baum et al., 2012; Edet et al., 2018). Both genomes of *Ae. triuncialis* (UC or CU) are nearly identical to the diploid genomes of *Ae. umbellulata* (U) and *Ae. markgrafii* (C) (Badaeva et al., 2004) and, unsurprisingly, the *Ae. triuncialis* cluster located between the diploid accessions with the U and C genomes. With the exception of *Ae. juvenalis* (DMU), which clustered with its D genome progenitor, polyploid species carrying a U genome were closely related to one another and to *Ae. umbellulata* despite having different non-U genomes (Badaeva et al., 2004; Kilian et al., 2011). Some of the wild relative species were sparsely represented in our collection, somewhat limiting our ability to establish clear relationships between the various genomes. Increasing their sample size is expected to refine this evolutionary relationship picture. Regardless, the collection had ample diversity to detect many putative new *Lr* genes.

The relationships observed in the phylogenetic tree were also observed by PCA. Overall, clustering patterns hinted at possible ascertainment biases; species of the A, B or D sub-genomes segregated more clearly, with few to no outliers, compared to other species. As the 90 K array consisted of SNPs previously discovered in cultivars of polyploid wheat, and its D genome progenitor *Ae. tauschii* (Wang et al., 2014), genotype calling may be limited to common alleles identified in the initial SNP discovery process (Albrechtsen et al., 2010). Although the genotyping data may not be sufficient to uncover novel ancestral relationships, it was nonetheless effective in revealing genetic variations at the species level and corroborating previously observed relationships (Bahrman et al., 1988; Badaeva et al., 2004).

### Detection of Previously Reported *Lr* Genes

A total of 13 QTNs identified were present within the mapped flanking markers of nine cataloged *Lr* genes. The QTN Excalibur_c21395_291 mapped between psr119 and mag3092, two markers tightly linked to *Lr28* (McIntosh et al., 1982; Sohail et al., 2014). Similarly, the QTN Excalibur_rep_c68362_62, mapped 1.6 Mb upstream, in a neighboring haplotype block of IWB41960, a marker tightly linked to the resistant gene *Lr18* (Dyck and Samborski, 1968; Carpenter et al., 2018). Both *Lr18* and *Lr28* loci QTNs were present within CC-NBS-LRR genes and showed significant allele-specific phenotypic differences, making them candidate genes worthy of further investigation.

Five QTNs were found to be in the same haplotype blocks as the cloned genes *Lr1* and *Lr34* and the genetic markers for *Lr16* (wmc764), *Lr32* (wmc43), and *Lr73* (wPt-4453) (McCartney et al., 2005; Cloutier et al., 2007; Krattinger et al., 2009; Thomas, 2010; Park et al., 2014). The QTNs close to *Lr1* and *Lr32* were present within CC-NBS-LRR and serine/threonine kinase domains, while the others were located within a 3-ketoacyl-CoA synthase domain or within genes of unknown function. These QTNs identified had within-block D’ statistics ranging from 0.54 to 0.85, where higher values suggest high linkage disequilibrium and similar association with phenotypic traits between pairs of SNPs in the same block (Cuyabano et al., 2014). Moreover, four of these five QTNs showed significant allele-specific phenotypic variation.

Overall, the lack of cloned genes or tightly linked markers restrict the ability to pinpoint the precise physical position of some *Lr* genes. QTNs linked to or within flanking markers of known *Lr* genes may serve as novel markers for gene cloning, however, fine-mapping, allelism tests, transformation genome editing (e.g., CRISPR) experiments must be performed to ascertain their identities.

### Identification of Novel Sources of Leaf Rust Resistance

The most prevalent class of known resistance genes encode intracellular immune receptors with NBS-LRR domains, many of which also possess a coiled-coil (CC) N-terminal motif. These genes play an important role in pathogen recognition and initiation of downstream signaling cascades. In the wheat genome, as many as 661 to 1,560 full-length NBS-LRR genes have been reported, higher than any other plant species (Gu et al., 2015; Steuernagel et al., 2020). Four of the six *Lr* genes cloned to date encode CC-NBS-LRR proteins (Feuillet et al., 2003; Huang et al., 2003; Cloutier et al., 2007; Thind et al., 2017).

GWAS for race-specific IT response and leaf rust severity identified a total of 16 QTNs within genes encoding complete CC-NBS-LRR domains. Of these, 11 explained greater than 5% of the phenotypic variation, while the remaining were small-effect loci. As discussed above, QTNs close to *Lr1*, *Lr18*, *Lr28*, and *Lr54* were in CC-NBS-LRR genes but the remaining were located where no known *Lr* genes have been mapped to date. For IT response, the most prominent QTNs within CC-NBS-LRR genes included D_contig18780_204 and Kukri_c19466_627, where for the former, all species in the U-genome group, SHWs and *Ae. tauschii* var. *strangulata* expressed the favorable allele, while for the latter, resistant accessions included wild relatives in the D-genome group and some SHWs, among others.

The highest number of QTNs within genes encoding CC-NBS-LRR proteins was identified in the spring panel rated for leaf rust severity. Of note is BS00065623_51 on the distal end of 7DS, where different subspecies of *T. aestivum*, such as *T. aestivum* ssp. *spelta*, was associated with the low-severity A allele, while most *T. aestivum* ssp. *aestivum* and all SHWs associated with the high-severity G allele. While the spring panel was made up of subspecies of *T. aestivum*, *T. turgidum* and SHWs, the winter panel was predominantly a collection of SHWs and wild relative species. The most notable leaf rust severity associated QTN identified in the winter panel may be Ex_c6145_2193. This QTN, present within a CC-NBS-LRR gene, was located on the distal end of the short arm of chromosome 1DS. Here, all the wild relatives including *Ae. crassa*, *Ae. juvenalis* and *Ae. cylindrica* among others, associated with the low-severity T allele, but the high-severity C allele was only detected in some *Ae. tauschii* and SHW accessions. Candidate CC-NBS-LRR genes identified here, in the primary as well as the wild gene pool, are valuable sources of genetic resistance.

Plant disease resistance is driven by complex mechanisms involving several layers of defense. Not surprisingly, the classes of known disease resistance genes have expanded greatly in the past few years. For leaf rust, in addition to *Lr34* encoding an ABC transporter (Krattinger et al., 2009), the cloned *Lr67* gene codes for a hexose transporter (Moore et al., 2015). Other pathogen resistance genes cloned in wheat encode serine/threonine protein kinases and wall-associated kinases (Cao et al., 2011; Shi et al., 2016). Identification of these diverse resistance proteins supports the possibility of uncovering novel classes of disease resistance genes. Consequently, in addition to those in CC-NBS-LRR genes, we identified a number of QTNs present in genes coding for other known resistance proteins in wheat and other plant species. A key QTN identified herein was Excalibur_rep_c67475_1759 located within a pleiotropic drug resistance-type ABC transporter protein, known to be involved in the secretion of fungal defense-related metabolites, including resistance to DON accumulation in wheat *Fusarium* head blight infection (Jasiński et al., 2001; Shang, 2009). Similarly, QTN Kukri_c39321_112 on 6B was associated with IT responses against three isolates (TDBG2, MBDS, and TJBJ). It was found within a gene encoding a ZTL-type beta-propeller/F-box domain protein known to regulate plant flowering time and provide resistance against yellow rust in wheat and powdery mildew in barley (Kim et al., 2005; Bozkurt et al., 2007; Dagdas et al., 2009). Here, species with the favorable allele included modern *T. turgidum* cultivars, *Ae. speltoides*, *Ae. sharonensis* and *T. timopheevii*, among others. These loci, located in novel genomic regions, are also recognized as putative candidate leaf rust resistance genes, and some may potentially confer resistance against multiple leaf rust isolates.

Overall, twice as many QTNs were identified in the spring panel as compared to the winter panel. This imbalance may be due to the difference in the number of accessions in each panel or the nature of the germplasm within each one where the spring panel comprised mostly the species used to design the wheat 90 K array, thereby providing higher quality genotyping. In addition, the potential for identifying novel disease resistance genes is also dependent on the mapping of the QTNs to the *T. aestivum* reference genome. As the reference only represents the A, B and D genomes of a single genotype, it may limit, but not prevent, our ability to identify rare resistance genes unique to the contrasting genomes of the wild relatives.

## Conclusion

The GWAS described herein highlights the multi-genic and complex nature of pathogen disease resistance where multiple markers were associated with different field environments and pathogen races. We identified several QTNs located near known *Lr* resistance genes providing, at the very least, novel markers for the cloning of these genes. Some of them were located within known resistance gene classes such as CC-NBS-LRR. As such, these become prime candidates for direct investigations. This study also identified novel leaf rust resistance loci from the domesticated *T. aestivum* and *T. turgidum* species that can be capitalized upon quickly, but also others from wild relative species that may be harnessed to add to the leaf rust resistance repertoire of wheat. Once cloned, the novel *Lr* genes can be transferred into adapted germplasm using modern genome-assisted breeding strategies, such as gene cassettes and genome editing (Wulff and Moscou, 2014; Wang et al., 2018). Gene cassettes allow multiple cloned disease resistance genes to be transformed simultaneously into a single genome to provide durable and broad-spectrum resistance, because the closely linked genes will not segregate, will be easy to select for, and will essentially have the advantages of gene pyramiding (Kolmer et al., 2009; Arora et al., 2019). Gene-specific markers can also be developed to facilitate the transfer of these genes through conventional breeding. The recently introduced CRISPR-Cas9 system in wheat (Zhang et al., 2016; Liang et al., 2017) offers many advantages. It can facilitate the investigation of candidate genes in any germplasm, bypassing the laborious fine-mapping experiments and enabling their functional analyses. We believe that gene editing could also be capitalized upon to “transfer” resistance genes from wild relatives through the allelic conversion of the orthologous domesticated alleles, providing that sufficient sequence similarity exists between the wheat and the wild relative alleles. This “long-shot” strategy would eliminate the need for the long, laborious and difficult introgression via crossing, and eradicate its associated linkage drag drawbacks. In conclusion, we described a powerful approach to identify QTN markers and candidate genes for leaf rust resistance through combining a broad germplasm including cultivated species and wild relatives, array-based genotyping, field severity and IT phenotyping and, through the use of several GWAS models.

## Data Availability Statement

The datasets presented in this study can be found in online repositories. The names of the repository/repositories and accession number(s) can be found in the manuscript/Supplementary Material.

## Author Contributions

FF performed the data analysis and wrote the manuscript; FY provided bioinformatics and statistics guidance; BM, SC and CP performed leaf rust severity phenotyping; BM performed race-specific phenotyping; CM and CH produced the wheat 90 K array data; GF co-developed the original experiment and provided some of the key germplasm; SC designed the experiments and co-wrote the manuscript. All authors read and edited the manuscript.

## Conflict of Interest

The authors declare that the research was conducted in the absence of any commercial or financial relationships that could be construed as a potential conflict of interest.

## Footnotes

1 https://plants.ensembl.org/Triticum_aestivum/Info/Index

2 https://wheat.pw.usda.gov/cgi-bin/seqserve/blast_wheat.cgi

## Supplementary Material

Supplementary Figure 1 Stacked bar plot summarizing genotype distribution of infection type response against six leaf rust isolates. Scores from the Stakman scale were converted to a linear 1–9 scale. Each bar represents the distribution of scores for a specific leaf rust isolate.

Supplementary Figure 2 Comparison between raw mean values and BLUP estimates for leaf rust severity. Scatter plots show mean values (y-axis) vs. BLUP estimates (x-axis) for overall severity in the spring **(A)** and winter **(B)** diversity panels. Boxplots show the interquartile range and median severity calculated using aggregate means **(C–E)** and BLUP estimates **(D–F)** for Morden (MDN), Ottawa (OTT) and Saskatoon (SK) in the spring diversity panel, and MDN and OTT in the winter diversity panel.

Supplementary Figure 3 Histograms describing the distribution of overall severity scores in the spring and winter panels. Raw mean values of the spring **(A)** and winter **(B)** diversity panels and genotypic best linear unbiased predictor (BLUP) estimates of the spring **(C)** and winter **(D)** diversity panels are illustrated. Genotypic BLUP estimates are conditional means which summarize phenotypic variation across all locations and years.

Supplementary Figure 4 Scree plots for estimating the K number of sub-populations in the SNP datasets for the race-specific infection type **(A)** and field leaf rust severity of the spring **(B)** and winter **(C)** diversity panels. PCAdapt and LEA are both PCA-based methods, where PCAdapt plots the number of PCs vs. proportion of variance explained, and LEA plots the Tracy–Widom statistic vs. K. Admixture detects population structure through trends in cross-validation error vs. K.

Supplementary Figure 5 LEA structure plots illustrating the ancestry mix of the subpopulations using K = 8 for the race-specific infection type **(A)**, K = 8 for leaf rust severity of the spring **(B)** and K = 6 for the winter **(C)** diversity panels.

Supplementary Figure 6 Box plots showing **(A)** percentage variance explained (r^2^) and **(B)** false discovery rate (FDR) adjusted *P*-values for quantitative trait nucleotides (QTNs) identified by each of the eight genome-wide association study (GWAS) models.

Supplementary Figure 7 Plot showing intersecting sets of quantitative trait nucleotides (QTNs) identified by each of the eight genome-wide association study (GWAS) models for all leaf rust traits. Names of the GWAS models are listed on the left side below the x-axis. Empty light-gray dots indicate GWAS models that are not part of the intersection, while filled black dots indicate GWAS models that are participating in the intersection. The size of each intersecting set is illustrated in the bar plot. Note that the intersection sets are disjointed, i.e., they are non-overlapping. For simplicity, only intersecting sets of size ≥ 5 are shown.

Supplementary Figure 8 Heatmap showing genotype calls for quantitative trait nucleotides (QTNs) that displayed significant allele-specific phenotype differences for **(A)** infection type response against six leaf rust isolates and leaf rust severity in the **(B)** spring and **(C)** winter diversity panel. Rows represent the entry number and genome symbol of the accessions in the germplasm and columns represent the QTNs. Genotypes AA represent homozygous alleles associated with low leaf rust infection type **(A)** or severity scores **(B,C)** and genotype BB represent homozygous alleles associated with high scores. Genotype AB and NA represent heterozygous alleles and no genotype calls, respectively. Different colors in the left panel represent the different groups of species in the germplasm.

Supplementary Figure 9 Rooted maximum likelihood phylogenetic tree illustrating the relationships between the wild *Aegilops*, *Triticum* and *Haynaldia* species in the germplasm. The leaf node labels are the entry number and genome symbol of each accession.

Supplementary Table 1 List of accessions included in the study and their respective genome, ploidy, growth habit, origin, and seed source.

Supplementary Table 2 Infection type scores of accessions rated for response against six leaf rust isolates. Scores are based on the Stakman scale **(A)** and their linear conversion using a 1–9 scale **(B)**.

Supplementary Table 3 Virulence and avirulence formulas for the six leaf rust pathogen isolates used to measure infection type response.

Supplementary Table 4 Genotypic and location-specific best linear unbiased predictor (BLUP) estimates for leaf rust severity of the spring **(A)** and winter **(B)** diversity panels.

Supplementary Table 5 List of known wheat leaf rust resistance genes, their source, gene and marker references, and position on the Chinese Spring reference genome v1.0 (IWGSC, 2018) mapped using gene sequences or flanking and linked markers.

Supplementary Table 6 Linear mixed model for leaf rust severity of the spring **(A)** and winter **(B)** panels across years and locations.

Supplementary Table 7 Average leaf rust severity of accessions of the spring **(A)** and winter **(B)** diversity panels calculated over all locations, years and replicates.

Supplementary Table 8 Chromosomal position of the wheat 90 K array SNP markers on the Chinese Spring exome sequence (ES) and reference genome sequence (CS) v1.0 (IWGSC, 2018).

Supplementary Table 9 List of eight accessions which did not cluster with other individuals of their respective species.

Supplementary Table 10 Significant quantitative trait nucleotides (QTNs) and linkage disequilibrium blocks (LDBs) for infection type (IT) response against six leaf rust isolates identified by association mapping using one single-locus and seven multi-locus models.

Supplementary Table 11 Significant quantitative trait nucleotides (QTNs) and linkage disequilibrium blocks (LDBs) associated with leaf rust severity in the spring and winter diversity panels as identified by association mapping using one single-locus and seven multi-locus models.

Supplementary Table 12 Functional annotation of genes located within 5 KB of quantitative trait nucleotides (QTNs) or peak single nucleotide polymorphism (SNP) markers of linkage disequilibrium blocks (LDBs) associated with infection type response against six leaf rust isolates and leaf rust (LR) severity of the spring and winter diversity panels.

Supplementary Table 13 Quantitative trait nucleotides (QTNs) located within flanking markers of leaf rust (*Lr*) resistance genes positioned onto the Chinese Spring reference genome sequence v1.0 (IWGSC, 2018) **(A)**, within the same linkage disequilibrium block (LDB) **(B)**, or a neighboring LDB **(C)**.
