# Supplementary figures and images for: Identification of New Leaf Rust Resistance Loci in Wheat and Wild Relatives by Array-Based SNP Genotyping and Association Genetics
Source: Front Plant Sci. 2020 Nov 16;11:583738. doi: 10.3389/fpls.2020.583738 (PMC7701059; doi:10.3389/fpls.2020.583738)

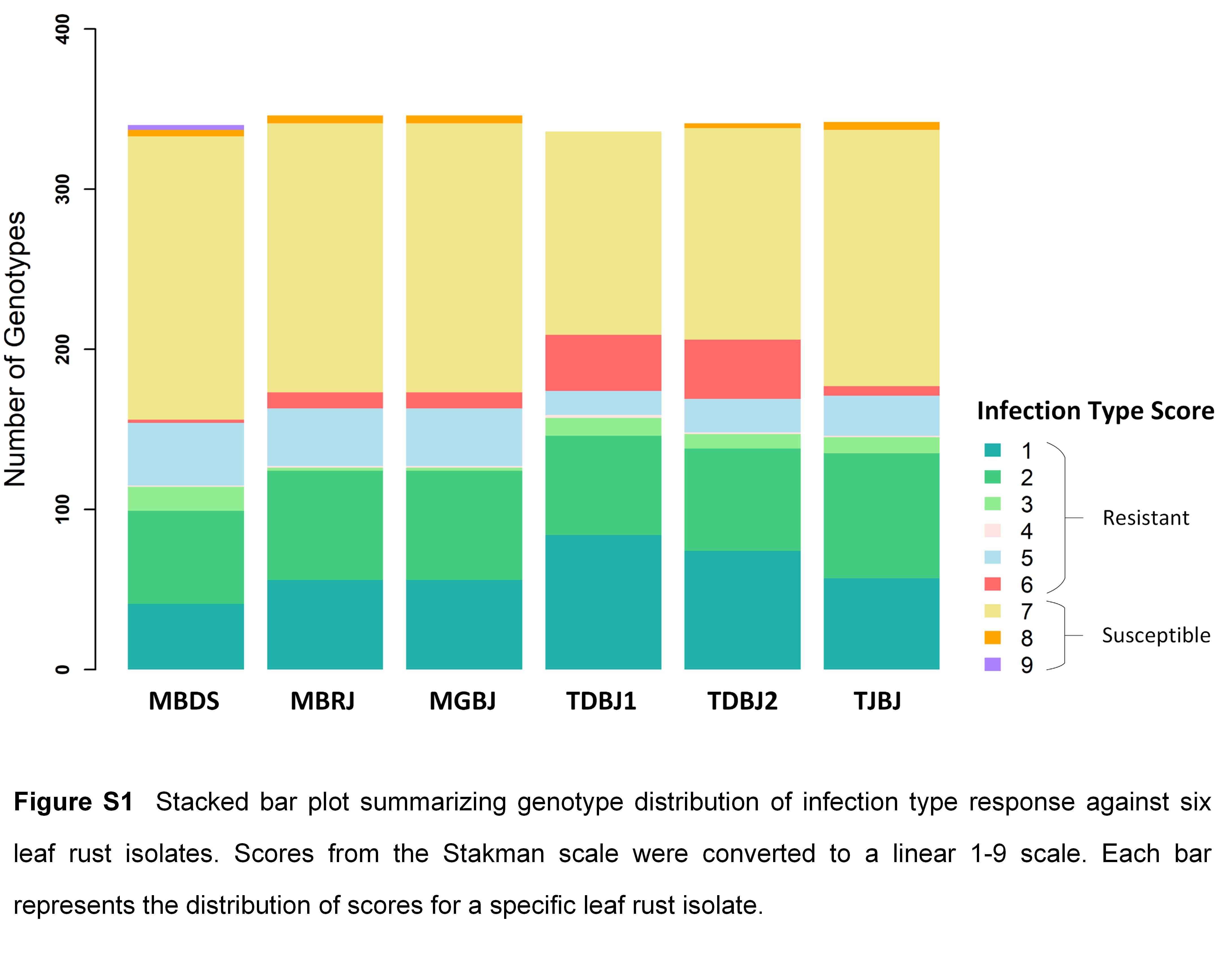

Supplement: Supplementary Figure 1 — Stacked bar plot summarizing genotype distribution of infection type response against six leaf rust isolates. Scores from the Stakman scale were converted to a linear 1–9 scale. Each bar represents the distribution of scores for a specific leaf rust isolate. [file Image_1.PNG]

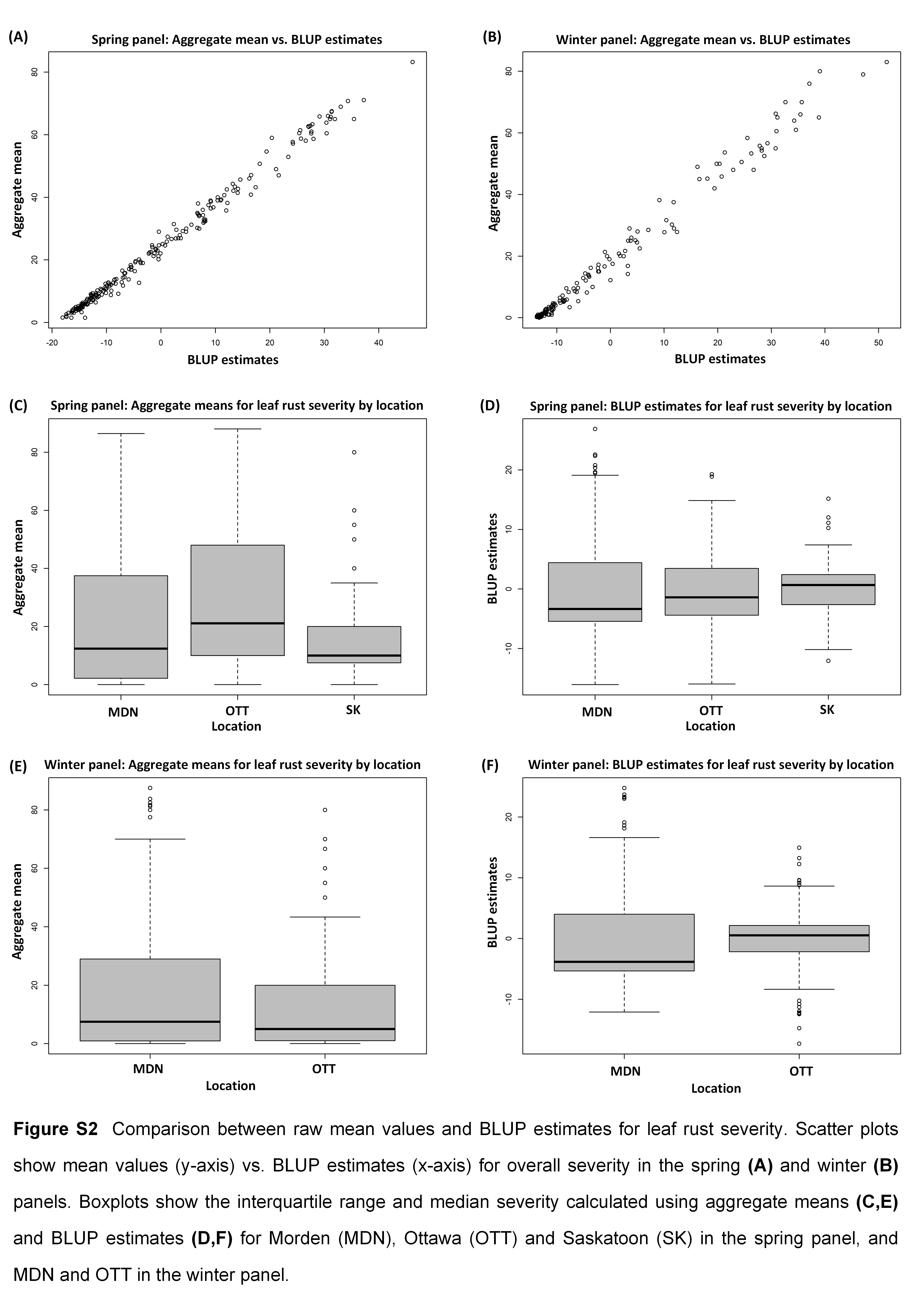

Supplement: Supplementary Figure 2 — Comparison between raw mean values and BLUP estimates for leaf rust severity. Scatter plots show mean values (y-axis) vs. BLUP estimates (x-axis) for overall severity in the spring (A) and winter (B) diversity panels. Boxplots show the interquartile range and median severity calculated using aggregate means (C–E) and BLUP estimates (D–F) for Morden (MDN), Ottawa (OTT) and Saskatoon (SK) in the spring diversity panel, and MDN and OTT in the winter diversity panel. [file Image_2.PNG]

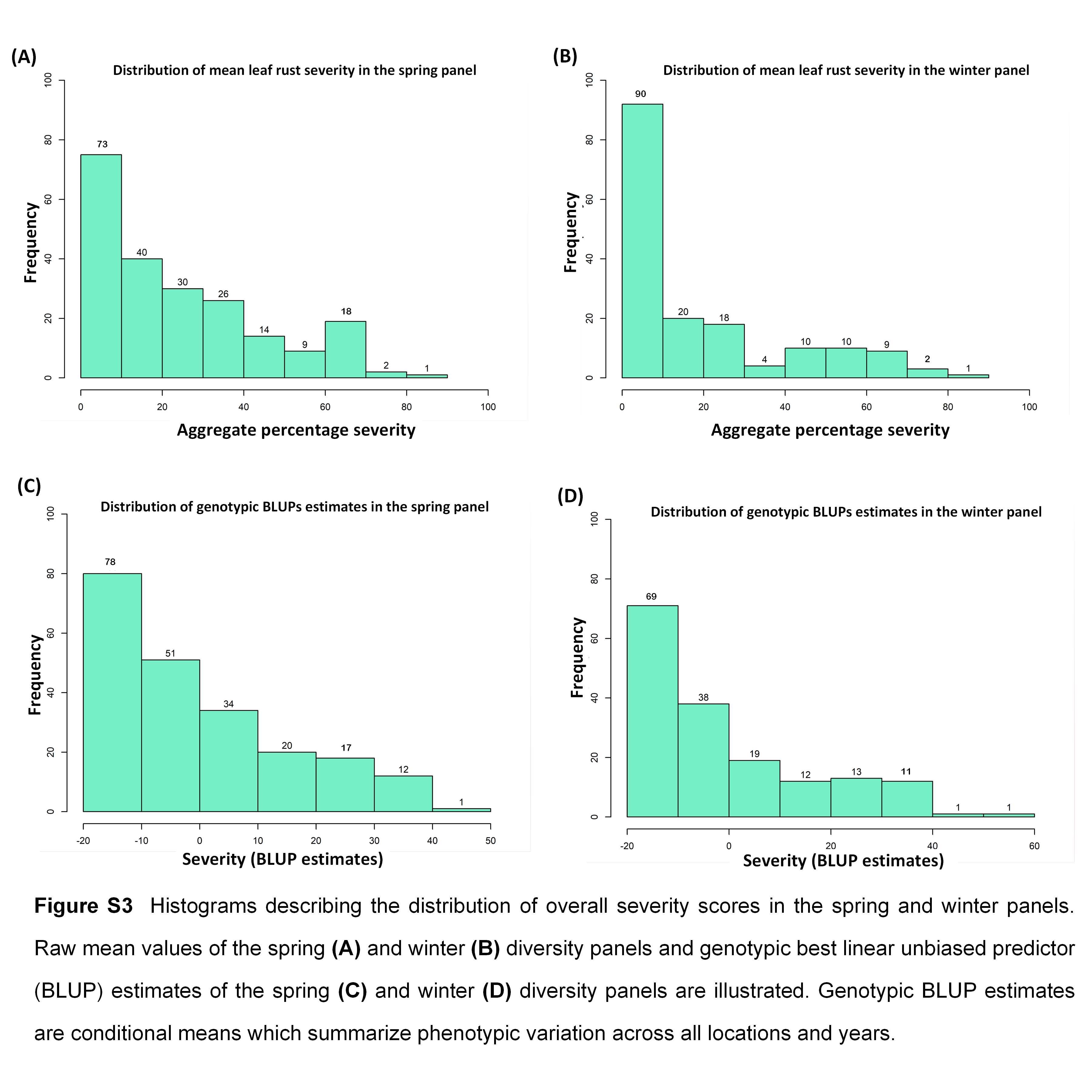

Supplement: Supplementary Figure 3 — Histograms describing the distribution of overall severity scores in the spring and winter panels. Raw mean values of the spring (A) and winter (B) diversity panels and genotypic best linear unbiased predictor (BLUP) estimates of the spring (C) and winter (D) diversity panels are illustrated. Genotypic BLUP estimates are conditional means which summarize phenotypic variation across all locations and years. [file Image_3.PNG]

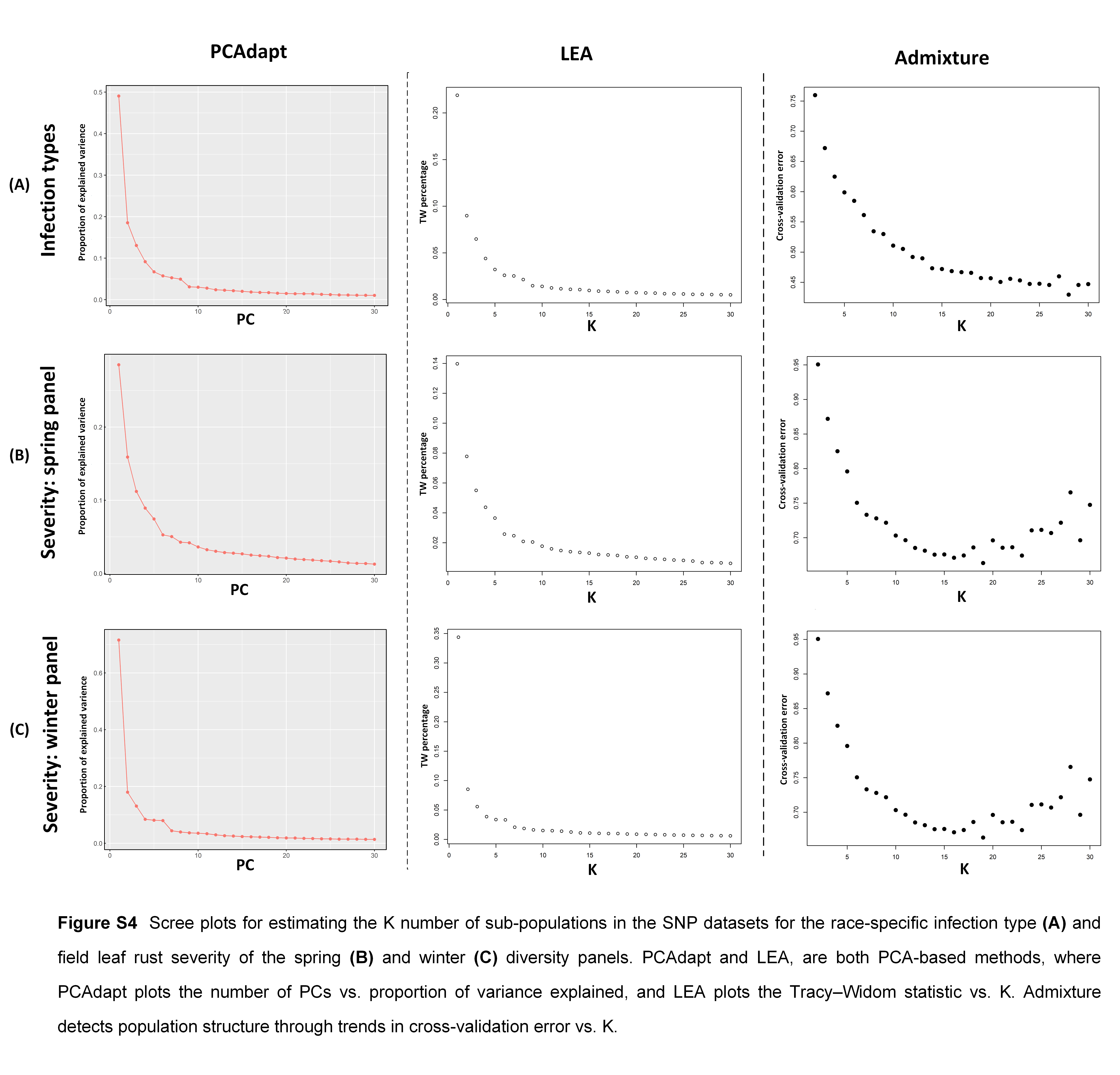

Supplement: Supplementary Figure 4 — Scree plots for estimating the K number of sub-populations in the SNP datasets for the race-specific infection type (A) and field leaf rust severity of the spring (B) and winter (C) diversity panels. PCAdapt and LEA are both PCA-based methods, where PCAdapt plots the number of PCs vs. proportion of variance explained, and LEA plots the Tracy–Widom statistic vs. K. Admixture detects population structure through trends in cross-validation error vs. K. [file Image_4.PNG]

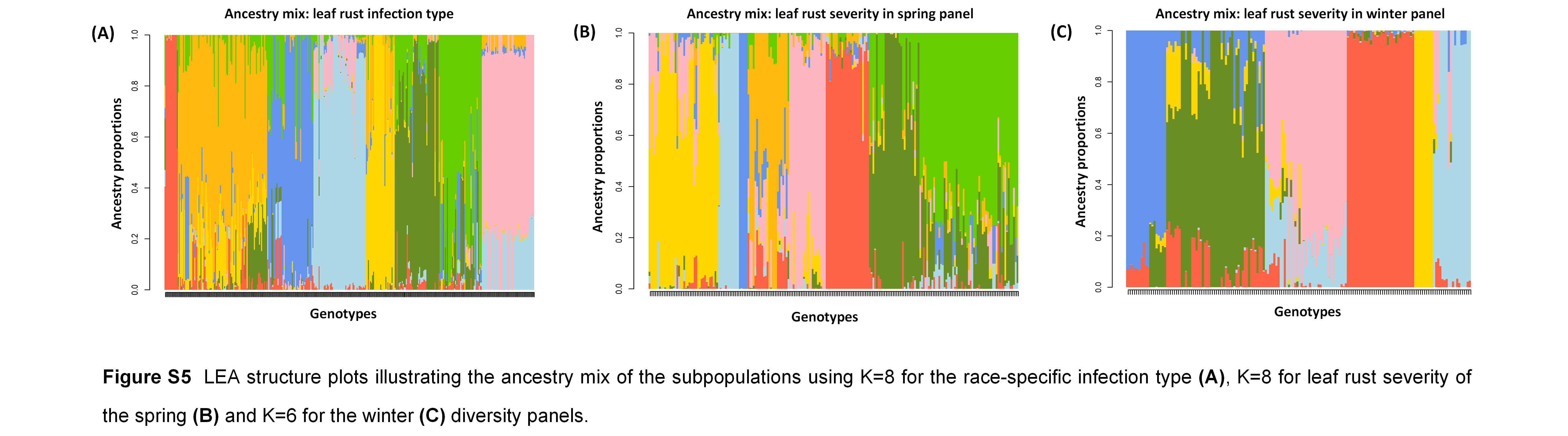

Supplement: Supplementary Figure 5 — LEA structure plots illustrating the ancestry mix of the subpopulations using K = 8 for the race-specific infection type (A), K = 8 for leaf rust severity of the spring (B) and K = 6 for the winter (C) diversity panels. [file Image_5.PNG]

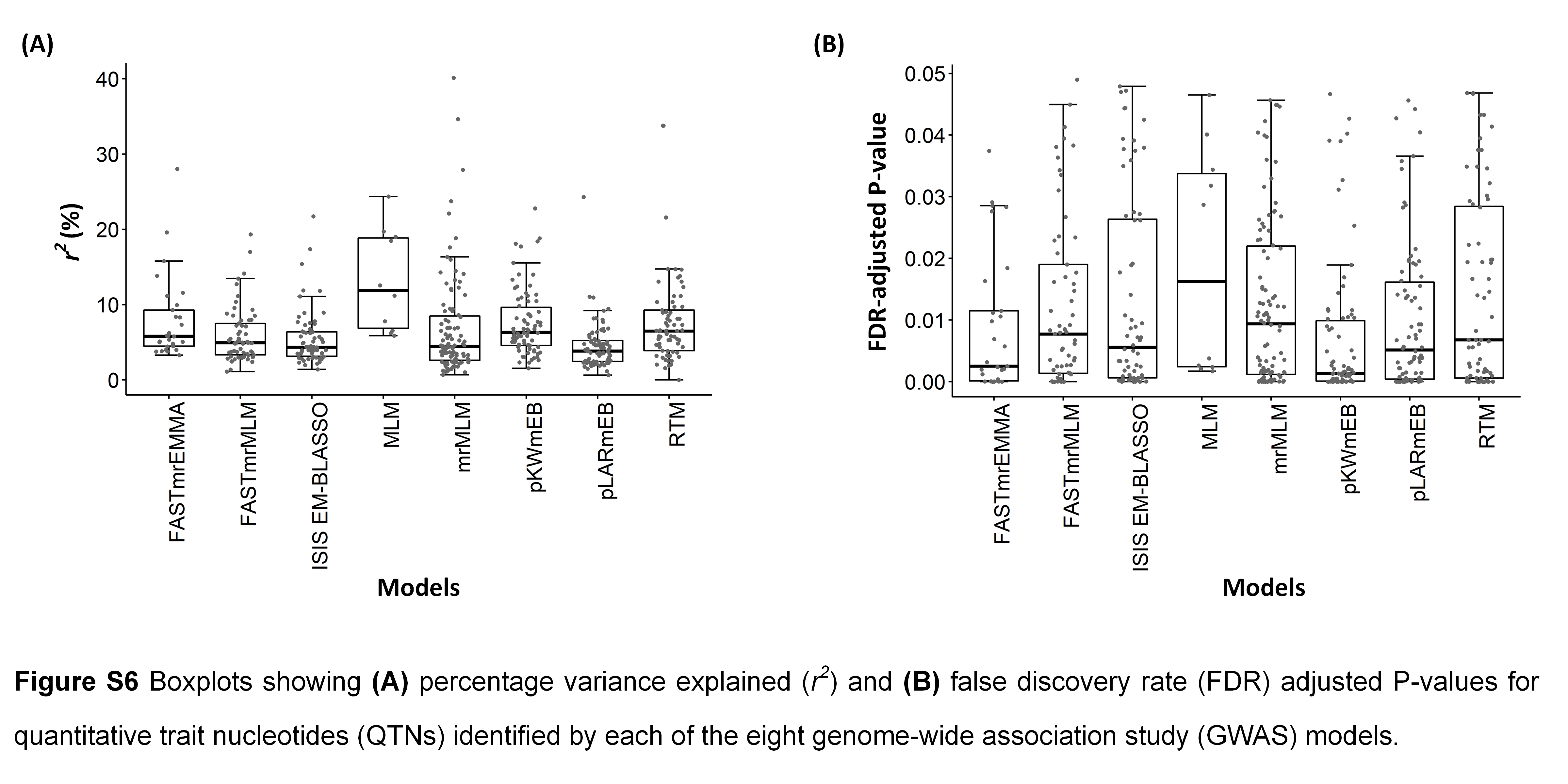

Supplement: Supplementary Figure 6 — Box plots showing (A) percentage variance explained (r2) and (B) false discovery rate (FDR) adjusted P-values for quantitative trait nucleotides (QTNs) identified by each of the eight genome-wide association study (GWAS) models. [file Image_6.PNG]

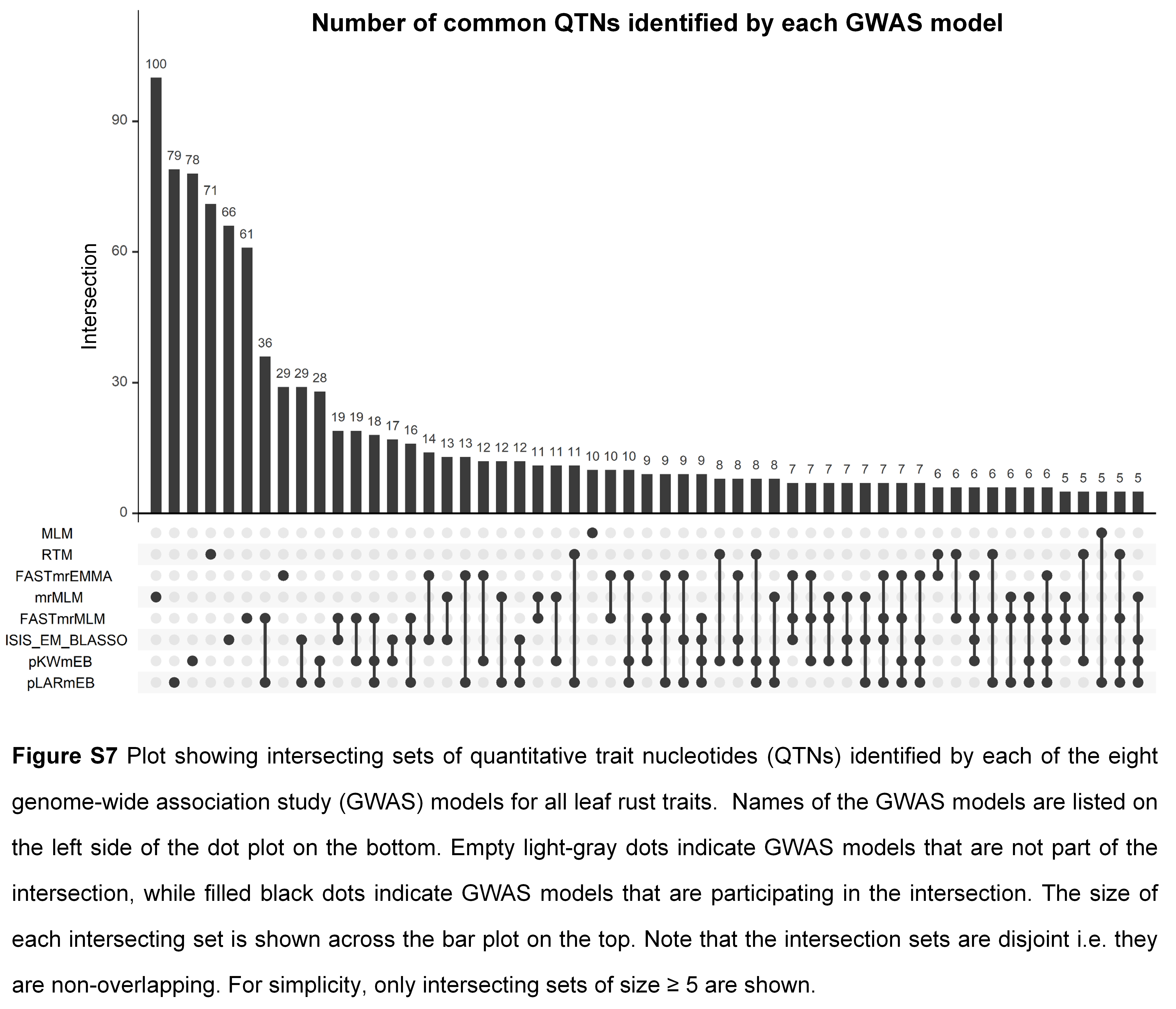

Supplement: Supplementary Figure 7 — Plot showing intersecting sets of quantitative trait nucleotides (QTNs) identified by each of the eight genome-wide association study (GWAS) models for all leaf rust traits. Names of the GWAS models are listed on the left side below the x-axis. Empty light-gray dots indicate GWAS models that are not part of the intersection, while filled black dots indicate GWAS models that are participating in the intersection. The size of each intersecting set is illustrated in the bar plot. Note that the intersection sets are disjointed, i.e., they are non-overlapping. For simplicity, only intersecting sets of size ≥ 5 are shown. [file Image_7.PNG]

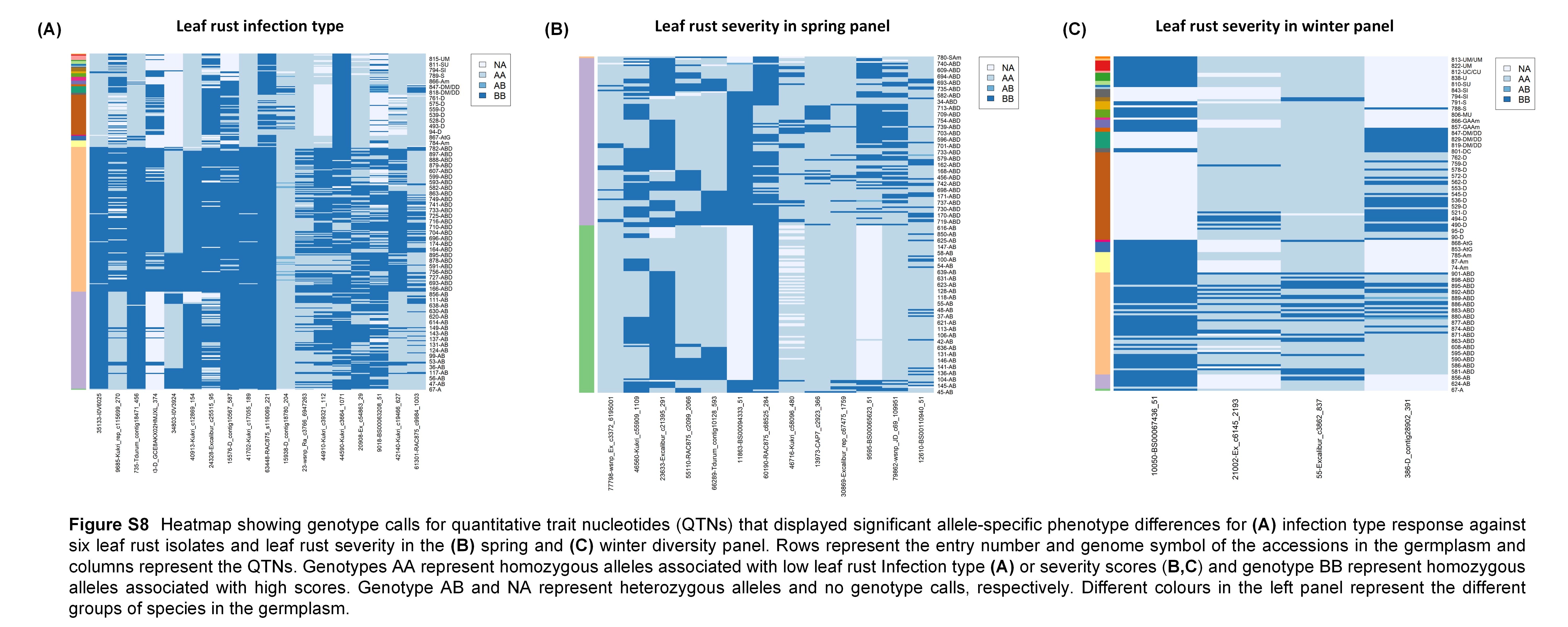

Supplement: Supplementary Figure 8 — Heatmap showing genotype calls for quantitative trait nucleotides (QTNs) that displayed significant allele-specific phenotype differences for (A) infection type response against six leaf rust isolates and leaf rust severity in the (B) spring and (C) winter diversity panel. Rows represent the entry number and genome symbol of the accessions in the germplasm and columns represent the QTNs. Genotypes AA represent homozygous alleles associated with low leaf rust infection type (A) or severity scores (B,C) and genotype BB represent homozygous alleles associated with high scores. Genotype AB and NA represent heterozygous alleles and no genotype calls, respectively. Different colors in the left panel represent the different groups of species in the germplasm. [file Image_8.PNG]

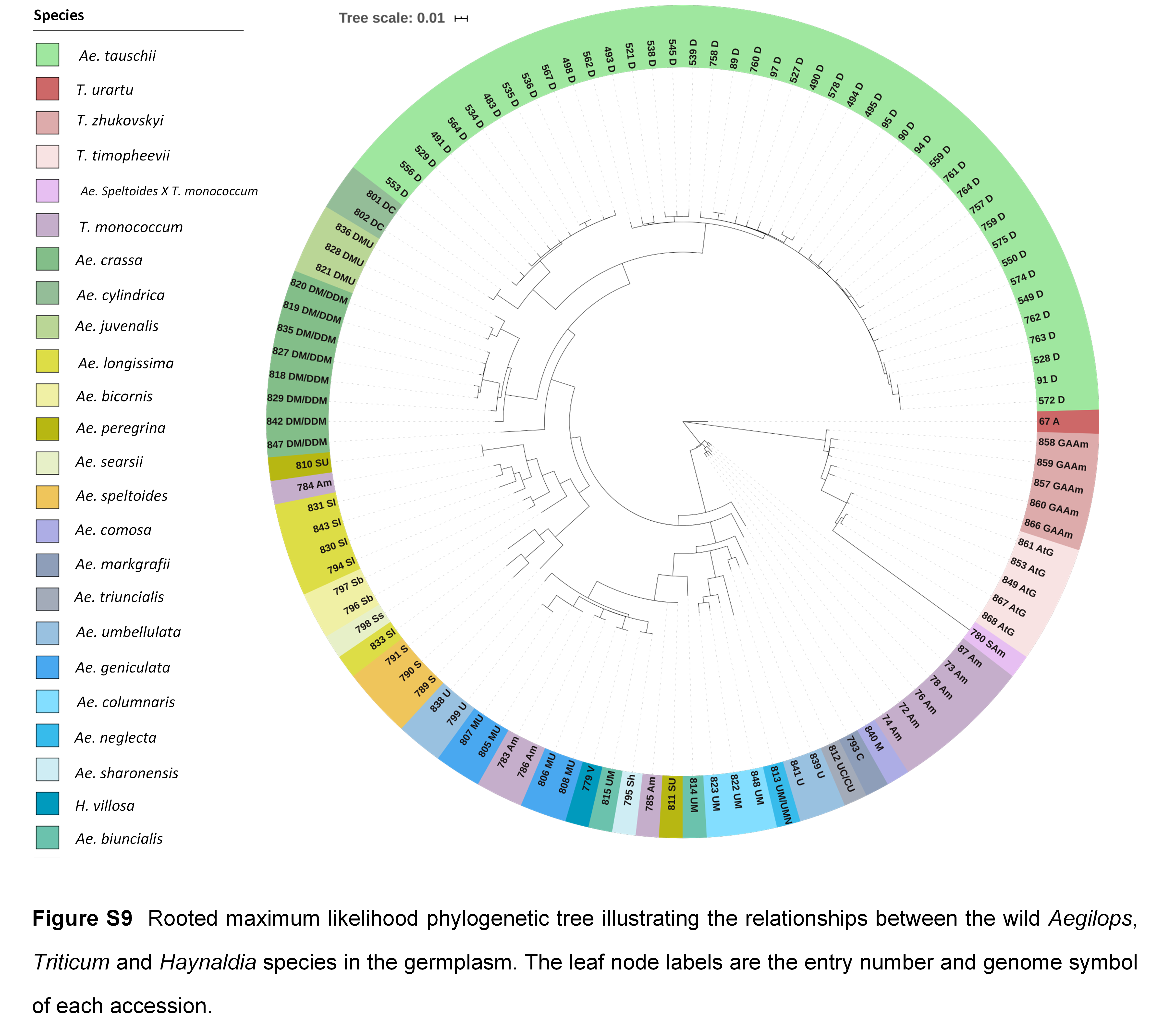

Supplement: Supplementary Figure 9 — Rooted maximum likelihood phylogenetic tree illustrating the relationships between the wild Aegilops, Triticum and Haynaldia species in the germplasm. The leaf node labels are the entry number and genome symbol of each accession. [file Image_9.PNG]
